# Nanostructured metal oxide semiconductor-based sensors for greenhouse gas detection: progress and challenges

**DOI:** 10.1098/rsos.201324

**Published:** 2021-03-10

**Authors:** Yogendra K. Gautam, Kavita Sharma, Shrestha Tyagi, Anit K. Ambedkar, Manika Chaudhary, Beer Pal Singh

**Affiliations:** Smart Materials and Sensor Laboratory, Department of Physics, CCS University, Meerut, Uttar Pradesh 250004, India

**Keywords:** greenhouse emissions, nanomaterials, metal oxide semiconductors, gas sensors

## Abstract

Climate change and global warming have been two massive concerns for the scientific community during the last few decades. Anthropogenic emissions of greenhouse gases (GHGs) have greatly amplified the level of greenhouse gases in the Earth's atmosphere which results in the gradual heating of the atmosphere. The precise measurement and reliable quantification of GHGs emission in the environment are of the utmost priority for the study of climate change. The detection of GHGs such as carbon dioxide, methane, nitrous oxide and ozone is the first and foremost step in finding the solution to manage and reduce the concentration of these gases in the Earth's atmosphere. The nanostructured metal oxide semiconductor (NMOS) based technologies for sensing GHGs emission have been found most reliable and accurate. Owing to their fascinating structural and morphological properties metal oxide semiconductors become an important class of materials for GHGs emission sensing technology. In this review article, the current concentration of GHGs in the Earth's environment, dominant sources of anthropogenic emissions of these gases and consequently their possible impacts on human life have been described briefly. Further, the different available technologies for GHG sensors along with their principle of operation have been largely discussed. The advantages and disadvantages of each sensor technology have also been highlighted. In particular, this article presents a comprehensive study on the development of various NMOS-based GHGs sensors and their performance analysis in order to establish a strong detection technology for the anthropogenic GHGs. In the last, the scope for improved sensitivity, selectivity and response time for these sensors, their future trends and outlook for researchers are suggested in the conclusion of this article.

## 1. Introduction

The whole world is craving for an environment on Earth plenteous of clean and fresh air. It ought to comprise reduced levels of greenhouse gases (GHGs); carbon dioxide (CO_2_), methane (CH_4_), nitrous oxide (N_2_O) and ozone (O_3_) and fluorocarbons. Human activities have increased the level of GHGs in the Earth's environment in dramatic ways over the past two centuries. Since the dawn of the industrial revolution in the early 1800s also 1900s, the burning of fossil fuels has greatly amplified the level of GHGs in the atmosphere, especially the CO_2_. Deforestation is considered as the second largest anthropogenic source of CO_2_ to the atmosphere [1]. The GHGs act like a blanket that absorbs infrared (IR) radiation and prevent it from escaping into outer space. This results in the gradual heating of Earth's atmosphere and surface and the process is named as global warming. The scientific community implicates that global warming might severely damage the Earth's atmosphere and climate.

Currently, most of the developing countries are struggling with the unstoppable excessive rate of change of climate in comparison to developed countries. The climate is changing and it is changing more quickly than is realized by us. According to a recent ‘Global Warming of 1.5°C’ special report of the Intergovernmental Panel on Climate Change (IPCC), the world has much less time before climate change becomes unmanageable. This report says that ‘climate-related risks for natural and human systems are higher for global warming of 1.5°C than at present but lower than at 2°C’ [2]. The IPCC fifth assessment report confirms that the total anthropogenic GHG emissions have continued to increase around 1970–2010 with larger absolute increases between 2000 and 2010 and still increasing, despite the growing number of climate change mitigation policies. It is quite evident that more than half of the observed increase in global average surface temperature from 1951 to 2010 was caused by the anthropogenic increase in GHG concentrations [3].

The scientific community has accepted the challenge of changing climate and is rigorously working towards the measurement and the quantification of this change. The prominent factors responsible for the volatility of the environment to a major extent are the GHGs. Diversified sensor technologies have been developed by various research groups worldwide for the detection of these environmentally hazardous gases. The existing sensor technologies are efficiently trying to develop suitable sensors for the precise monitoring of the environment deteriorating GHGs. The different gas detection technologies include mainly metal oxide semiconductor (MOS)-based sensors, catalytic gas sensors, electrochemical gas sensors, optical gas sensors and acoustic gas sensors, etc. The performance of every sensor is based on some characteristic including sensitivity, selectivity, detection limit, operating temperature, response time and recovery time, etc.

Among many gas sensing technologies, nanostructured metal oxide semiconductor (NMOS) based gas sensors have shown excellent performance over other available sensors because of their excellent physical and chemical properties and unique structure [4–10]. These materials have a wide band gap, allowing them to have a full spectrum of electronic properties. The properties of the NMOSs are greatly affected by the material's size, microstructure and moreover the inclusion of some impurities like metals and ions etc., drastically improves their electrical/optical properties [11]. NMOS-based sensors have been shown to be sensitive to a large range of GHGs, mainly to CO_2_, CH_4_, N_2_O and O_3_, with excellent responses varying with both target gas concentration and device operating temperature [12]. The past four decades have seen incredible research in the development of MOS-based gas sensors which includes the production of innovative materials and associated fabrication technologies, and of course improved efficiency of sensors [13]. These studies lead to the good quality and efficient portable gas sensing devices, which have shown excellent sensitivities and selectivity; efficient fast response/recovery time; low operating temperature or even the ability to function independent of temperature which would lead to efficient power consumption; stability in the performance of the sensor in environment conditions; and minimum use of the chemically sensitive layer. All these features would allow the sensors to be used a multiple number of times for measurements [14]. However, there is a need for further improvements in their performances such as accuracy, selectivity and reliability in order to meet today's requirement of precise monitoring of the GHGs [15]. There have already been a lot of studies carried out by researchers globally on the topic of NMOS gas sensing materials in general and few of them excellently describe the gas sensing mechanism in depth. These studies conclude several strategies for the further improvement in the gas sensing properties of the NMOS sensors from the perspective of gas sensing mechanisms [16–18].

In the next part of the introduction, we briefly discuss the current concentration of the major GHGs present in the troposphere of the Earth's atmosphere and reported by several research agencies worldwide and their effects on human life with a view to emphasize the need for accurate detecting/measuring NMOS-based tools for the GHGs.

### 1.1. Concentration of greenhouse gases in the Earth's environment

**Figure 1.**
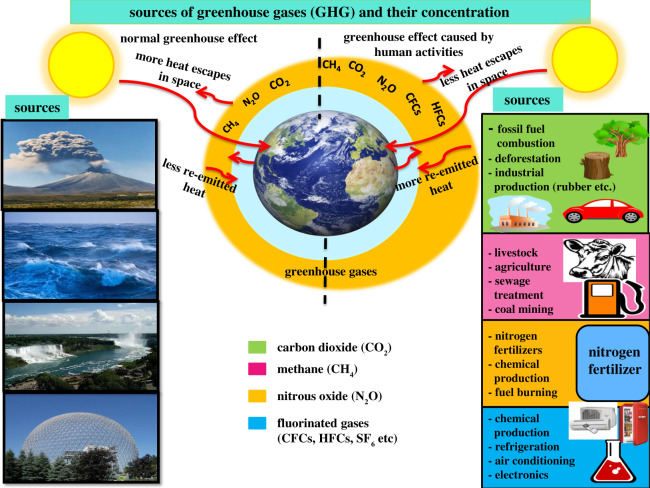
A comparative diagram of natural greenhouse gases and the variety of human activities contributing towards the increasing concentration of greenhouse gases in the Earth's environment.

GHGs trap the Earth's emitted radiation, which might escape back to space and when these gases are present in the appropriate amount in earth's environment, keep the earth warm enough to sustain life on it. The primary GHGs in the atmosphere are water vapour, CO_2_, CH_4_, N_2_O and O_3_. GHGs specifically trap photons of wavelengths in the IR region and therefore act as important temperature regulators of our atmosphere. These gases differ in their ability to absorb energy, that is, they have various radiative efficiencies. They also differ in their atmospheric residence times. Water vapour accounts for by far the largest greenhouse effect (90–85%) because water vapour emits and absorbs IR radiation at many more wavelengths than any of the other GHGs, and there is much more water vapour in the atmosphere than any of the other GHGs. Although water vapour content is highly variable to measure and owing to lack of proper measurements of global water vapour, it is not predictable how much content of water vapour gives rise to the greenhouse effect that in turn affects the temperature of Earth. GHGs have increased greatly since the preindustrial times owing to human various actions as can be seen in figure 1.

The emission of anthropogenic CO_2_, as an end product of carbon-fuel combustion, is mainly related to the consumption of fossil fuels, construction materials, minerals and industrial materials. The water solubility of CO_2_ decreases with the increase in temperature of water and owing to this attribute more CO_2_ given off into the atmosphere. However, owing to its high solubility and reactivity, CO_2_ allows ready exchange of itself between oceans and atmosphere. CO_2_ has a very long residence time in the atmosphere, its emissions cause increases in atmospheric concentrations of CO_2_ that will last thousands of years [19].

**Figure 2.**
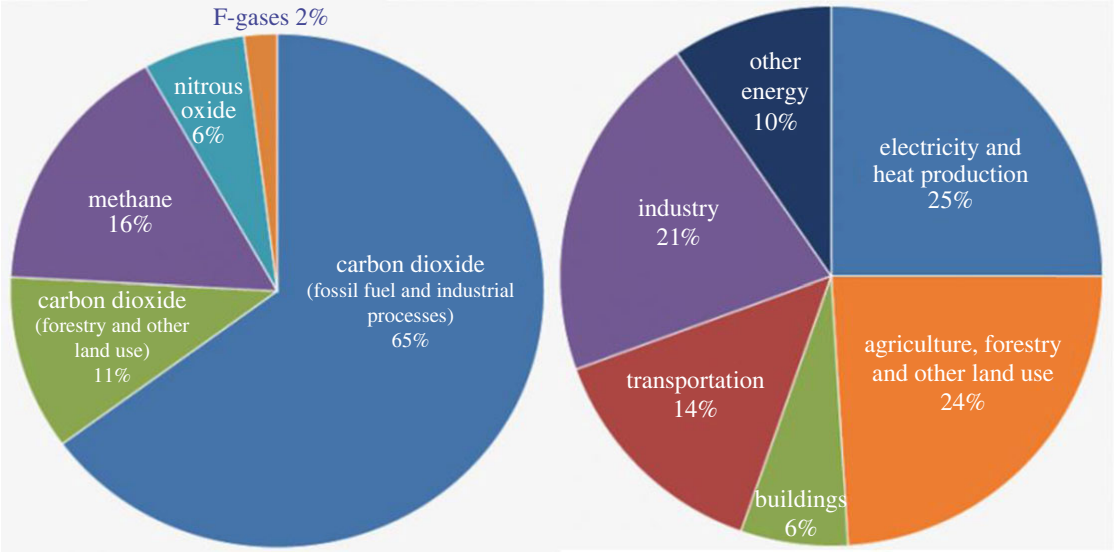
The current global concentration of greenhouse gases in Earth's environment along with their global economic sector wise contribution [20].

The present global concentration of various GHGs in the environment along the economic sector wise contribution is listed in table 1 and shown in figure 2. As is evident from table 1, CO_2_ is considered to be the biggest contributor for climate change as it is present in the highest amount in the Earth’s environment, i.e. 76% [21]. The power sector may be considered as the major contributor to its high level of concentration and the industrial sector emerges as the second major contributor. The global concentration of CH_4_ is 16%. This is emitted from the production and transport of coal, natural gas and oil. CH_4_ emission also takes place from the decomposition of organic waste in agriculture, in municipal solid waste, landfills and the raising of livestock. The contribution of N_2_O emission is 6% to the global GHG emissions. This is supposed to be emitted during agriculture and industrial activities, as well as during the combustion of solid waste and fossil fuels. The chloroflorocarbon (CFC) contribution is of 2% to the global GHG emission.

**Table 1.** Current concentration of greenhouse gases in the environment along with their sector wise contribution [20].

s. no	greenhouse gases (GHGs)	total concentration of GHGs in atmosphere (%)	sector wise contribution in GHG concentration					
			power sector (%)	industrial sector (%)	transportation sector (%)	commercial and residential sector (%)	agriculture sector (%)	other energy (%)
1.	CO_2_	76	25	21	14	6	24	10
2.	CH_4_	16						
3.	N_2_O	6						
4.	fluorinated gases	2						

In order to have a clear picture on the causes of emission of major GHG's and their role in the global greenhouse effect starting from CO_2_, CH_4_, N_2_O, O_3_ to CFCs are briefly discussed in the next subsections.

#### 1.1.1. Concentration of carbon dioxide gas

According to the World Meteorological Organization's Greenhouse Gas Bulletin, universally the middle value of the concentration of CO_2_ is found to arrive at 407.8 parts per million (ppm) in the year 2018, whereas it was 405.5 (ppm) in 2017. This trend uncovers a consecutive decline of the atmosphere owing to the CO_2_ concentration level. This increment of CO_2_ concentration level is attributed to fuel burning, backwoods fires, volcanic emissions and unpredictable natural mixes. The clear decision to combat this issue is to lessen the use of petroleum products, incrementing the use of non-polluting powers, start forest preservation endeavours and prevent volcanoes from ejecting [22,23].

#### 1.1.2. Concentration of methane gas

CH_4_ is another significant gas promoting the greenhouse effect. Its expansion is incredibly changing the climatic condition. It assimilates the sun's heat and warms the environment. It is considered to be multiple times more intense than CO_2_. It is created by the common deterioration of rice paddies, bogs, the guts of creatures, the spoiling of waste and the dissemination of petroleum products like coal, oil or gas [24]. CH_4_ is broadly used for power generation, hydrogen and ethylene production, and domiciliary warming. It is exceptionally unpredictable in nature and when blended in with the air, may handily cause an eruption in closed zones. Early identification of the existence of CH_4_ gas is critical to prevent blasts in mechanical and household applications [25].

#### 1.1.3. Concentration of nitrous oxide gas

The anthropogenic sources of N_2_O emission include; agriculture fields, fuel combustion, wastewater management and industrial processes. On the whole, these are increasing the amount of N_2_O in the atmosphere. The natural source of N_2_O emission in the Earth's atmosphere is its nitrogen cycle. In the nitrogen cycle, there is the natural circulation of nitrogen among the atmosphere, plants, animals and microorganisms that live in soil and water. These microorganisms break down nitrogen in soils and the oceans. The N_2_O molecules may reside in the atmosphere for an average of 114 years before being removed/destroyed through chemical reactions. The aftermath of 1 pound of N_2_O molecules on warming the atmosphere is around 300 times that of 1 pound of CO_2_ [26]. Globally, almost 40% of the total N_2_O emissions come from various human activities [27].

#### 1.1.4. Concentration of fluorocarbons

The group of fluorocarbons, as is described in the Kyoto Protocol, can be referred as hydrofluorocarbons (HFCs), perfluorocarbons (PFCs) and sulfur hexafluoride (SF6) [28]. The group of HFCs is particularly broad. The concentration levels of these fluorocarbons have increased substantially over recent decades. Their contribution to climate forcing is currently still limited, although steadily increasing from 0.5% in 1990 to 0.8% in 2004 and 1.5% in 2017. Their contribution is expected to continuously increase in the near future because of the long lifetimes (greater than 1000 years in some cases) and the increase in the emissions of new HFCs, such as HFC-134a [29].

The status of GHGs in the environment emphasizes the superabundant need for the centralization of these gases in the Earth's environment with the end goal of developing sensors.

### 1.2. Severe impacts of greenhouse effect on human life

**Figure 3.**
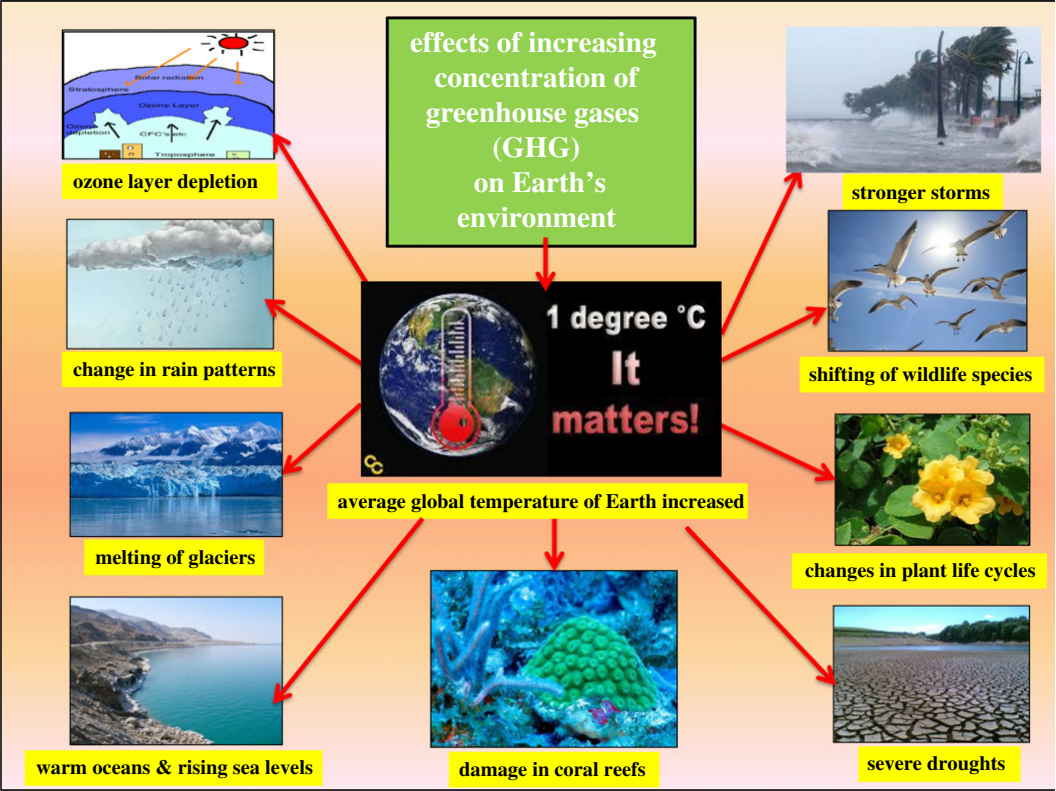
A schematic diagram of major severe consequences of increased concentration of GHGs in earth's environment.

Figure 3 depicts the schematic view on some of the severe consequences of increased concentration of GHGs in the Earth's environment created by various human activities such as burning fossil fuels, industrial production (rubber, plastic, etc.), deforestation, sewage treatment, air conditioning, refrigeration, etc. As is evident from figure 3, the first direct well-known effect of the increased concentration of GHGs is the increase in the global average temperature of the Earth. According to the global climate report issued by National Oceanic and Atmospheric Administration, USA. (NOAA), the first three months of the current year 2020 were characterized by warmer-than-average conditions across much of the globe. The January–March 2020 temperatures for Europe and Asia were expected to be the highest in the 111-year record [30]. This has far-ranging environmental and health impacts on living conditions of human life of our planet. Some of the major issues imposed by this are discussed below in short.

#### 1.2.1. Ozone layer depletion

It is known that the O_3_ layer on the Earth protects us all from the sun's harmful radiation, but the increased concentration of GHGs have damaged this shield. Less O_3_ layer means low protection from ultraviolet (UV) light which may over the time, damage crops and lead to higher skin cancer and cataract rates. The O_3_ hole is actually a region of exceptionally depleted O_3_ in the stratosphere prominently over the Antarctic region or the South Pole as is reported in the Scientific Assessment of Ozone Depletion 2014. Primarily it was noted in the beginning of 1970s. On the basis of historical record, it is found that the total column O_3_ values of less than 220 Dobson Units were not observed prior to 1979. So, 220 Dobson Units of O_3_ is generally used as the boundary and the values below this can be used for representing the O_3_ loss. This report also suggests that the O_3_ depletion is not limited to the area over the South Pole but occurs over the latitudes that include North America, Europe, Asia, and much of Africa, Australia and South America. The ozone depleting-substances (ODS) include CFCs, hydrochlorofluoro carbons (HCFCs), carbon tetrachloride and methyl chloroform. Although ODS are emitted at the Earth's surface, they are eventually carried into the stratosphere in a process that can take as long as 2–5 years [31].

#### 1.2.2. Change in rain patterns

Changes in global precipitation are among the most important and least well-understood consequences of climate change or increasing GHG concentrations. Climate change is altering the rain fall patterns globally. According to research [32] climate change affects the global precipitation patterns and shifts them across the land and ocean. The climate model of this research predicts that the addition of heat-trapping gases in the atmosphere will shift precipitation in two main ways. The first shift is in a strengthening of existing precipitation patterns. This is commonly called wet get wetter, dry get drier. The second shift is a change in storm tracks, which should move away from the equator and toward the poles as atmospheric circulation changes. Increases and decreases in the frequency and magnitude of river flood events may vary by region.

#### 1.2.3. Melting of glaciers and rising sea levels

Global researchers in recent years have found and reported that the world's glaciers are now in the smallest shape they have been in human history [33]. Latest research quantifies how much the world's lost glaciers have contributed to rising sea levels for example; the sea levels rise by 3 mm each year and the oceans warm further. Scientists estimate that this thermal expansion will force sea levels up even more. It is also claimed that some glacial regions in Europe, Canada, the US and New Zealand could see their glaciers completely disappear by 2100. This fact is also stated in the fifth Assessment Report of the Intergovernmental Panel on Climate Change (IPCC, 2014). According to this report, during the period 1901–2010 the global average sea level rose 19 cm. It is estimated that by 2100 the sea level will be between 15 and 90 cm higher than it is now and will threaten 92 million people [34]. Including this, many extreme temperature conditions are becoming more common. Since the 1970s, unusually hot summer days (highs) have become more common over the last few decades in various parts of globe. Record-setting daily high temperatures have become more common than record lows. The decade from 2000 to 2009 had twice as many record highs as record lows [34].

#### 1.2.4. Damage to coral reefs

Coral reefs are considered as one of the most diverse ecosystems on this planet. They offer a surplus of benefits both to natural ecosystems and to the human population. Coral reefs bring in enormous funds to coastal countries through tourism, fishing and discoveries of new bio chemicals and drugs, and additionally they provide natural coastal protection and building materials [35]. However, coral reefs are experiencing massive die-outs all around the world. At first, many thought the biggest threats to coral reef health were direct anthropogenic effects such as water pollution and sedimentation, but now it is clear that the 50–70% of coral reefs are directly affected by anthropogenic global climate change [36]. Rising global temperatures, increasing oceanic CO_2_, and other consequences of climate change are all affecting coral reef health in a negative way.

#### 1.2.5. Stronger storms

Hurricanes are the most violent storms on the planet, and strong storms are getting stronger because of the warmer oceans. Stronger storms are known to us by different names, for example hurricanes in the Atlantic Ocean, typhoons in the western Pacific Ocean or cyclones in the Indian Ocean. These storms arise owing to a small atmospheric disturbance located in or near a tropical ocean. If water temperatures are warm enough, and atmospheric conditions are supportive with moisture and uniform winds, a tropical system can evolve. In the Atlantic, the system first becomes a tropical depression. As it gets stronger the system matures to a tropical storm and then finally, when winds rise over 74 mph, it turns into a hurricane. Climate scientists claimed that slower steering currents resulting from a warmer climate may have contributed to cyclonic activity [37]. According to this report, the tropical storm activity in the Atlantic Ocean, the Caribbean and the Gulf of Mexico has increased during the past 20 years.

#### 1.2.6. Shifting of wildlife species

Change in the Earth's temperature and other impacts of climate change are severely affecting animal species. The world has witnessed many of them go extinct every year owing to changing ecosystems and habitat loss, particularly the tigers, the giant panda bears, green turtles, Asian elephants, polar bears and penguins among others as is stated by Marike Lauwrens in a blog [38]. Climate change affects animal species in a number of ways; the changing climate has made their habitat less comfortable, and sometimes even inhospitable. They have to deal with increases in water, air and solid waste pollution that affects the food they eat and the surroundings they live in. Animals also experience habitat loss because these animals have to alter their breeding and feeding patterns in order to survive.

#### 1.2.7. Change in plant's life cycle

The largest known economic impact of climate change is upon the agriculture productivity and livelihood of the large population living in developing countries [22]. Owing to global warming, the expected decline in agricultural productivity is assumed to be by 9–21% in developing countries [39]. In northern and western parts of Asia, rice and maize productions have been reduced to great extent, with an increase in CO_2_, N_2_O and CH_4_ gas emissions by 5% from 1998 to 2011 [40]. Research studies concluded that a small increase in temperature had larger effect than elevated CO_2_ on grain quality and the rising trend of global warming is considered to be more striking than precipitation over the twentieth century [41]. The rise in CO_2_ concentration has both positive and negative effects on agricultural productivity. Through increased photosynthesis, CO_2_ is predicted to have beneficial physiological effects. C3 crops such as wheat and rice have a higher impact of increased CO_2_ than C4 plants such as maize and grasses. Above the threshold level of CO_2_, the hinderance with the respiration system of plants leads to slow metabolic processes resulting in low productivity.

#### 1.2.8. Droughts

Global warming is having a profound impact on the processes of soil degradation and is contributing to the desertification of the driest areas on the planet. Desertification destroys all the biological potential of affected regions, turning them into barren and unproductive land. As recognized by the United Nations (UN) on the occasion of the World Day to Combat Desertification in 2018, 30% of land has been degraded and lost its real value [42].

Apart from this, the UN's Food and Agriculture Organization (FAO) states that climate change is raising serious doubts about food availability in its report on the state of world food and agriculture. It warns that a decline in agricultural production would result in food shortages, most severely affecting sub-Saharan Africa and South Asia. Moreover, the World Health Organization (WHO) states that global warming will cause infectious diseases such as malaria, cholera or dengue to spread to many more areas of the planet. On the other hand, extreme heat will increase and aggravate cardiovascular and respiratory problems [43].

In the view of the above facts and alarming signs, it is the need of the hour to monitor/detect the anthropogenic emissions of GHGs in Earth's atmosphere with utmost priority worldwide. Here, monitoring alludes to the confirmation of the existence of GHGs and their quantification. Therefore, this review article presents a comprehensive analysis of the various studies available on NMOS-based GHG sensors for CO_2_, CH_4_, N_2_O and O_3_ along with the factors which affect their sensitivity, selectivity and the stability. Furthermore, the performance of NMOS-based GHGs sensors is critically reviewed and compared with recent studies on other gas sensing techniques such as optical gas sensor, chemiluminescence, photoacoustic spectroscopy, gas chromatography and electrochemical sensors, etc. In the next section of this review paper, different gas sensing technologies have been widely discussed, with a special highlight on MOS-based gas sensors and its sensing mechanism being elaborated in detail.

## 2. Gas sensors and their classification

A gas sensor is a device that detects the presence of the volatile substances in vapour phase, both qualitatively and quantitatively (concentration) in a specific volume [44]. The gas sensors are classified on two different basis: (i) type of sensing material: optical absorption [45], catalytic, thermo conductive [46], solid electrolytic [47] and MOSs [48,49], and (ii) sensing mechanism: gas sensors categorize on detection method is additionally divided into two groups; (a) variation in electrical parameters and (b) sensing mechanism based on other types of alterations like; optical, catalytic, thermometric, photoacoustic, chemiluminescence and gas chromatographic, etc. Materials, such as metal, MOSs, polymers and carbon-based materials are used as gas sensors depending on the change in electrical properties in the presence/absence of target gases [50].

### 2.1. Chemical gas sensors

Chemical sensors were defined by the International Union of Pure and Applied Chemistry (IUPAC) in 1991. A chemical sensor is a device that transforms chemical information, ranging from concentration of a specific sample component to total composition analysis, into an analytically useful signal. It is considered as the official definition of these sensors [51]. There is a change in properties like composition, pH, concentration, etc. In these sensors, effective signal recognition, reception and transduction form the base of quantitative investigation using chemical sensors.

### 2.2. Optical gas sensors

The optical gas sensors are based on the detection of change in physical properties such as intensity, emission spectra, colour, polarization and velocity of light, etc., which are caused by absorbance, reflectance, fluorescence or scattering of light of a particular wavelength by the gaseous species [52]. The use of an optical gas sensor is limited owing to high cost and difficulty in miniaturization [50].

### 2.3. Electrochemical gas sensors

This type of gas sensor consists of electrochemical cells that are made from at least two electrodes, one is a sensing electrode or working electrode and the other is a reference electrode and these electrodes are connected through a thin layer of electrolyte. Electrochemical sensors permit the diffusion of gases through a membrane to the working electrode where oxidation or reduction of the gases takes place [52]. The electrochemical sensors can be divided in three groups: (i) potentiometric sensors, (ii) amperometric sensors, and (iii) conductometric sensors. In potentiometric electrochemical sensors, the resultant potential of the electrode or membrane is measured while in amperometric sensors, the variation in resulting current is measured. On the other hand, for conductometric sensors, frequent series of conductivities of the electrode are measured.

### 2.4. Catalytic gas sensor

Catalytic sensors have been used for the detection of combustible gases for almost a century. The majority of metal oxides and their compounds have catalytic properties. Combustible gas mixtures do not burn until they have reached a certain ignition temperature, but the gas will start to combust even at lower temperatures in the presence of a specific chemical process. This procedure is known as catalytic combustion. The sensor based on this catalytic principle is called a catalytic gas sensor. To test the output of the catalytic gas sensor, the Wheatstone bridge is used. Pellistor and thermoelectric are the two types of catalytic gas sensor [53].

### 2.5. Mass sensitive gas sensor

In the mass sensitive sensor, the target gas mounts on the sensitive adsorbing layer because of which the mass of the sensor surface changes. Surface acoustic wave (SAW) based sensors micro-cantilever and quartz crystal microbalance (QCM) are examples of mass sensitive sensors. The variation in mass shows the change in the properties of sensitive material [54]. The first mass sensitive sensor was based on the estimation of bulk acoustic waves (BAW) in a piezoelectric quartz gem resonator which is sensitive to mass changes. In these types of sensors, acoustic sensors are widely used because their detection mechanism is a mechanical or acoustic wave. When the acoustic wave propagates through the material, a notable variation in the characteristics of the wave (amplitude/velocity) propagation is observed. Changes in velocity can be observed by estimating the frequency and phase characteristics of the sensor and can then be correlated to the corresponding physical quantity being measured. The receptor and transducer are the main components of an acoustic wave sensor. A receptor is an element which is sensitive to an analyte while the transducer is an element which converts the response into an electrical signal [53].

### 2.6. Magnetic gas sensors

The change in the paramagnetic properties of the analyte is the basic principle of magnetic sensors [55]. These sensors are represented by a certain type of oxygen monitor because oxygen has a high magnetic susceptibility as compared to other gases [56]. Therefore, a gas sensor based on the paramagnetic principle and Pauli's exclusion principle for oxygen can be built with only minor cross sensitivities.

### 2.7. Thermometric gas sensors

In thermometric sensors, chemical reactions take place after the interaction of gases with the surface layer of the sensor; these chemical reactions become the cause of variation in temperature. This variation is indicated as the variation in electrical signals such as current, voltage and resistance. It has a temperature probe coated with a chemically selective layer. The device operates by detecting the heat transfer during the catalytic reaction between the coating at the sensor's surface and analyte and the related variation in temperature inside the device is measured [52].

### 2.8. Photoacoustic spectroscopy

Gas phase spectroscopy is now very popular in a wide range of fields such as atmospheric, chemistry, biology, medical sciences and safety. This spectroscopy is based on the photoacoustic effect which was first reported by Alexander Graham Bell [57]. The spectroscopic gas sensors have proved to be invaluable tools. There are various ways of using gas sensors, and different demands are placed on each application. For one particular gas compound, some applications need very high sensitivity, while others benefit more from a sensor that can calculate a wide range of gases or benefit from a miniaturized footprint. There is also a desirable time resolution, as well as selectivity, robustness and little or no need for sample preparation and maintenance; a significant number of these criteria are fulfilled by photoacoustic spectroscopy and its sensors [58].

### 2.9. Chemiluminescence

Chemiluminescence (CL) is one of the luminescent phenomena that can be described as the emission (UV, Vis or IR) emitted by a chemical reaction. In recent years, there has been a growing development in the application of CL to chemical analysis, particularly. As an analytical tool, CL detection is well known for its high sensitivity, quick response and absence of unwanted luminescence in the background. It can occur in the gas, liquid or solid phase, thus facilitating and expanding its scope of analytical applications [59].

### 2.10. Gas chromatography

Gas chromatography (GC) is an analytical technique that is widely used for quality control and additional detection and quantitation of components in a mixture, GC is often used as a tool where the identification of very small amounts is taken into account. It is a common method of chromatography used to segregate and investigate the gas or liquid that can be vaporized without disintegration as a part of analytical science [60].

### 2.11. Metal oxide semiconductor-based gas sensors

Over the past decades, different types of sensors have been developed by using various sensing materials and different transduction stages [61]. MOS-based gas sensors stand apart from others because of their easy underlying mechanism and lower cost [62]. Various hazardous gases such as CO, CO_2_, NH_3_, N_2_O, SO_2_, O_3_, LPG, CH_4_, etc. are detected successfully and efficiently by using MOS-based sensors. In general, MOSs such as SnO_2_, ZnO, In_2_O_3_, TiO_2_, CeO_2_, Fe_2_O_3_, WO_3_, CdO, CuO, etc. are used for the detection of GHGs. There are numerous techniques to synthesize NMOSs for GHGs sensors such as chemical vapour deposition, hydrothermal, co-precipitation, sol-gel method, pulsed laser deposition (PLD), radio frequency (RF) and direct current (DC) sputtering, thermal evaporation, etc. [63,64].

The essential materials used as a gas detector incorporate MOSs, characteristic conduction polymers, conductive composites polymers, metal oxide/composite polymers and other new materials. These materials can be used on various transduction units, also called chemiresistive SAWs, QCM, optical transducer and MOS field-effect transistor (MOSFET). Based on observations, the chemiresistive MOS has great potential to be used for novel gas sensors [50]. The chemiresistor is the easiest and smallest in the size estimation method with a straightforward detection mechanism. There are two primary sorts of MOS-based sensors including n-type whose larger part charge bearer is an electron (such as tin dioxide (SnO_2_), titanium dioxide (TiO_2_), zinc oxide (ZnO), iron oxide (In_2_O_3_), etc.), and p-type whose majority charge transporter is hole (such as cobalt oxide (CoO), nickel oxide (NiO), etc.) [65]. Potential uses of these chemical sensors incorporate natural inspections, and aviation vehicle wellbeing observation and several others [66].

**Figure 4.**
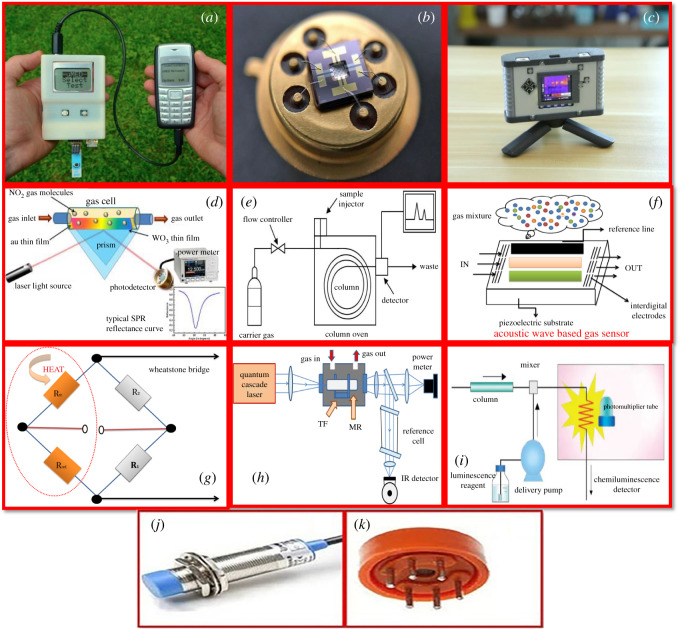
(*a*) Electrochemical sensor in the form of device [67], (*b*) MOS sensor [68], (*c*) thermal sensor, in the form of camera [69], (*d*) optical sensor [70], (*e*) gas chromatography, (*f*) mass sensitive sensor, (*g*) catalytic sensor, (*h*) photoacoustic spectroscopy, (*i*) chemiluminescence, (*j*) magnetic sensor, and (*k*) chemical sensor.

Chemiresistive (MOS-based) gas sensors show change in resistance upon exposure to the gases by the oxidative interactions with the negatively charged chemisorbed oxygen [64]. The gas sensing mechanisms such as, gas reaction, reaction rate and selectivity are significantly affected by the surface nature, porosity, microstructure of the detecting material, the existence of catalysts and the sensing temperature [67]. Generally, the operating temperature and thickness of the film affects the response of MOS-based sensors. The response to a specific gas can be incredibly boosted by including a catalytic metal to the oxide layer, however, unnecessary stacking can decrease the response [68]. The sensitivity and selectivity to the specific gases are also affected by the grain size of the oxides because grain boundaries act as an electron scattering centre [69]. Different types of gas sensors are shown in figure 4 and table 2 briefs the transduction mechanism and principle of the various gas sensors.

**Table 2.** Different types of gas sensors and their advantages and disadvantages.

type of sensor	measured quantities	principle	advantages	disadvantages
MOSs-based sensors	conductivity	conductometric	wide range of target gases, fast response, low cost and long lifetime	high-energy consumption, sensitivity to environmental factors, non-selective
electrochemical gas sensor	charge, current, voltage, resistance, inductance, etc.	potentiometric, amperometric, resistive, etc.	can measure toxic gases in low concentration	easy contamination
magnetic gas sensor	magnetic flux density, magnetic moment, etc.	paramagnetic	consumes low power, relatively affordable	sensitivity to environmental factors
thermometric gas sensors	temperature, specific heat, heat flow, etc.	calorimetric	easy to operate in absence of oxygen, low cost and adequate sensitivity for industrial detection	risk of explosion, intrinsic deficiencies in selectivity
catalytic gas sensor	temperature, resistance	catalytic/gas oxidation	simple, low cost, measures flammability of gases	requirement of air or oxygen to work
chemical gas sensor	composition, concentration, pH, etc.	changes in properties	simple design and low cost	cross sensitivity of other gases, limited temperature range
optical gas sensor	light intensity, wavelength, polarization, etc.	fluorescence, optical, etc.	simple operational process in absence of oxygen, unaffected from electromagnetic interference	high cost and difficulty in miniaturization
mass resistive gas sensor	change in the characteristics such as amplitude and velocity	acoustic	long lifetime and avoiding secondary pollution	sensitive to environmental change
gas chromatography	mobile phase (gas and liquid)	partition co-efficient	high sensitivity and selectivity	high cost, difficulty in miniaturization for portable applications
chemiluminescence	photocurrent/dark current	emission of radiation	high sensitivity, quick response	nonlinear behaviour
photoacoustic spectroscopy	absorbed electromagnetic energy	photoacoustic effect	high sensitivity	stability, miniaturization, integration and selectivity

#### 2.11.1. Gas sensing mechanism

The change in electrical conductivity or resistivity of MOSs is the basic principle of gas detection in MOS-based gas sensors [71,72]. In MOS, under the normal atmospheric conditions and typical operating temperature, an electron-depleted surface layer is developed in the presence of atmospheric oxygen that is adsorbed or chemisorbed on the surface. At first, oxygen is consumed by the metal oxide surface when the surface layer is exposed to air, i.e. the oxygen ionic species $O_{2}^{-}$, O^−^ and O^2^ get adsorbed on the top of the metal oxide grains. This will prompt a band twisting, and a depletion region called the space charge field is formed. When the target gas particles arrive at the surface of the metal oxide grains, they will interact with the oxygen anions and change the concentration level of electrons in the metal oxide substances. This will result in a change in conductivity, thereby providing an electronic response signal that can be measured. The detection mechanism in metal oxide gas sensors is identified with ion sorption of species over their surfaces. At the point when the gas sensor is exposed to oxygen, the adsorbed oxygen particle will be framed with the oxygen atoms removing the electrons from the metal oxide inside. The following reaction ((2.1)–(2.4)) steps show the adsorption kinematics [73–75]:

2.1 $$O_{2}(\mathrm{gas})\Leftrightarrow O_{2}\;(\mathrm{absorbed}),$$

2.2 $$O_{2}(\mathrm{absorbed})+e^{-}\Leftrightarrow O_{2}^{-},\;(<100^{\circ }C),$$

2.3 $$O_{2}^{-}+e^{-}\Leftrightarrow 2O^{-}\;(100-300^{\circ }C),$$

2.4 $$\mathrm{and}\;\;\;\;\;\;\;\;O^{-}+e^{-}\Leftrightarrow O^{2-}(>300^{\circ }C).$$

The types of chemisorbed oxygen ions are determined by the operating temperature of gas sensors. For temperatures below 100°C, between 100°C and 300°C and more than 300°C, the oxygen ions are $O_{2}^{-}$, O^−^ and O^2−^, respectively [76].

**Figure 5.**
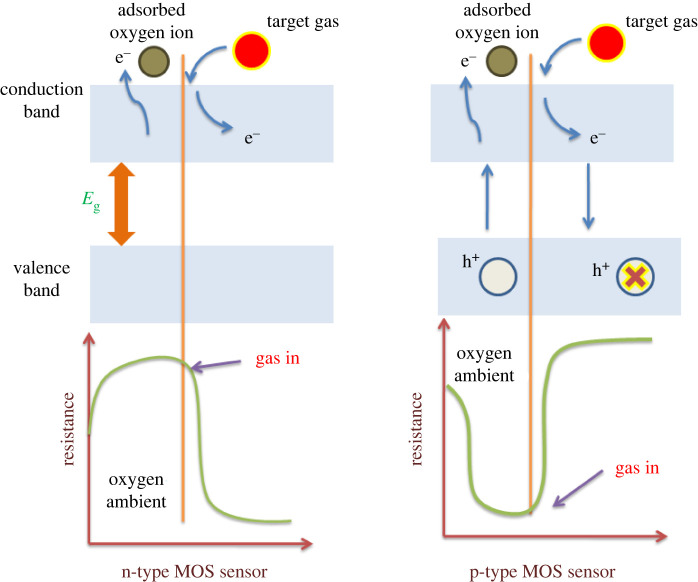
Schematic diagram: sensing mechanism of n-type and p-type MOSs.

Naturally unsafe gases can be segregated into two classes depending on the oxidizing and reducing impacts. NO_2_, NO, N_2_O and CO_2_ gases fall into the category of oxidizing while H_2_S, CO, NH_3_, CH_4_ and SO_2_ gases in reducing. When an n-type MOSs gas sensor is exposed to oxidizing gas, the target gas reacts with the ambient oxygen ions and retains the electrons at the surface. It reduces electron concentration in MOSs. Because electrons are the majority charge transporters in MOSs, the conductance of n-type MOSs decreases on exposure to oxidizing gas. In the case of a p-type MOSs gas sensor, holes are the majority charge transporters. The concentration of holes inside the MOSs is increased owing to the extracted electron. So, it implies that on the exposure of oxidizing gases, the conductance of p-type MOS increases. The schematic diagram for the sensing mechanism of n-type and p-type MOSs is shown in figure 5.

The following oxidizing reactions (equations (2.5)–(2.8)) show the reaction path between metal oxide and oxidizing gases [74–80]:

2.5 $$N_{2}O\;(\mathrm{gas})+e^{-}\to N_{2}O^{-}(\mathrm{ads})$$

2.6 $$N_{2}O^{-}(\mathrm{ads})\to N_{2}(\mathrm{gas})+O^{-}(\mathrm{ads})$$

2.7 $$CO_{2}(\mathrm{gas})+e^{-}\to CO^{2-}(\mathrm{ads})$$

2.8 $$\mathrm{and}\;\;\;\;CO^{2-}(\mathrm{ads})+O^{-}(\mathrm{ads})+2e^{-}\to \mathrm{CO}(\mathrm{gas})+2O^{2-}(\mathrm{ads})$$

For n-type MOSs, the gas sensing response can be defined by the following equation:

2.9 $$S^{n}=\frac{\;R_{g}}{R_{a}},$$

while the response of gas for p-type semiconductor oxide to oxidizing gas is generally defined by the following equation:

2.10 $$S^{p}=\frac{R_{a}}{R_{g}}.$$

Here, *R_g_* and *R_a_* are the electrical resistances of the sensor estimated in the presence of gas and net dry air, respectively. The sign of resistance changes upon the presence/absence of oxidizing and reducing gases for n-type and p-type MOS-based sensors is given in table 3.

**Table 3.** The type of resistance changes upon presence/absence of oxidizing and reducing gases for n-type and p-type MOS-based sensors.

type of sensitive material	type of target gas	resistance change	response
n-type	oxidizing	resistance increase	*S* = *R*_g_/*R*_a_
n-type	reducing	resistance decrease	*S* = *R*_a_/*R*_g_
p-type	oxidizing	resistance decrease	*S* = *R*_a_/*R*_g_
p-type	reducing	resistance increase	*S* = *R*_g_/*R*_a_

#### 2.11.2. Gas sensors parameters

##### 2.11.2.1. Response

The resistance of the sensor is altered when the sensor is exposed to a gas. The change in resistance of a gas sensor in the air and in the target gas as a function of analyte gas concentration is defined as response. The above equations (2.9) and (2.10) define the response behaviour for n-type MOSs and p-type MOSs, respectively.

##### 2.11.2.2. Selectivity

This is the measurement of the ability of the gas sensor to differentiate one gas among the mixture of gases at the same concentration level.

##### 2.11.2.3. Sensitivity

This is concerned with the magnitude of change in electrical resistance owing to species of the target gas.

##### 2.11.2.4. Limit of detection

Under the given conditions, the capability of the sensor to detect the least possible concentration of the analyte gas is defined as the limit of detection (LOD) of the sensor.

##### 2.11.2.5. Working temperature or operating temperature

The temperature at which the gas sensor shows the supreme response for a certain concentration of the analyte gas is known as the working temperature for a gas sensor.

## 3. Greenhosue gas sensors

### 3.1. Metal oxide semiconductor-based carbon dioxide gas sensors

The effect of CO_2_ on global warming is very large. So it is necessary to detect and control its emission and CO_2_ sensors can play a significant role to observe and control indoor air quality. These sensors are affordable, very sensitive and can work at room temperature. These sensors can be easily implemented in agricultural applications, the Earth's atmosphere, etc. and are nature friendly and user friendly as well. CO_2_ sensors play a vital role in the food processing industry as a preservative, in medical care as breath analysers and in biotechnology as environmental incubators [81].

A large number of investigations on metal oxide materials have been reported for CO_2_ detection, the summary of various MOS-based CO_2_ gas sensors is given in table 4. The table includes detail of various CO_2_ sensors based on pure MOSs and metal-doped MOSs, MOS-based composites and hetrojunctions with their synthesis methods and sensor parameters like; operating temperature (°C), response/recovery time (*t*_res_/*t*_rec_) and LOD, etc. The ZnO nanorods with and without adding a catalyst ZnSn(OH)_6_ (ZHS) microcubes have been synthesized on a p-type silicon substrate by a simple hydrothermal method and it is found that ZHS has a good ability to be used as a catalyst. In this report, ZHS microcubes can enhance the sensing performance of CO_2_ by 35% in comparison to previously reported studies [104]. Ca-doped ZnO thin films coated Langasite (La_3_Ga_5_SiO_14_) substrate have been prepared which shows good sensing performance towards CO_2_ at high temperature by using a SAW sensor. Maximum CO_2_ sensing is found to be 25 000 ppm with 2.469 kHz response at 400°C [88]. CO_2_ sensors based on ZnO nanorods of length (1–3 µm) doped with Ge, Nd and W, etc. were synthesized by using the mechanochemical combustion method. The doped ZnO nanorods were found to be more sensitive than the undoped-ZnO under an air atmosphere [94]. Owing to the inclusion of Na doping, the surface of plane ZnO film turned to a wrinkle network having granular structure. This nanostructured 2.5% Na-doped ZnO film showed high sensitivity (81.9%) for CO_2_ gas in comparison to 1.0% for pure ZnO film. The recovery and response time of Na-doped ZnO was also increased owing to the doping of Na atoms [91]. Multifunctional ZnO thin film shows maximum sensitivity of 400 ppm at 350°C and good response and recovery time of 75 and 180 s, respectively, towards CO_2_ gas [90]*.* Organic materials featuring ethynylatedthiourea derivatives have been developed to act as active materials for the detection of carbon dioxide. The resistance value of these derivatives was found to increase when it was exposed to CO_2_ gas [93].

**Table 4.** Summary of various MOS-based carbon dioxide (CO_2_) gas sensors. (C = concentration; *t*_res_/*t*_rec_ = response time/recovery time; LOD = limit of detection; response is defined as *R*_a_/*R*_g_ (for reducing gases) or *R*_g_/*R*_a_ (for oxidizing gases), *R*_a_: resistance of the sensor exposed to air, *R*_g_: resistance of the sensor exposed to the target gas.)

material	structure	synthesis method	target gas	C (ppm)	operating temp. (°C)	response	*t*_res_/*t*_rec_	LOD	ref.
BiOCl–Au	nanoparticles	surfactant-assisted	CO_2_	400	300	63.2	1.3/1.5 s	100 ppm	[82]
Ba/SmCoO_3_	powders	aqueous solution	CO_2_	—	373	∼1.5	202 s	—	[83]
Zn/SnO_2_	thin films	spray pyrolysis	CO_2_	500	300	90	55/82 s	—	[84]
LaOCl/SnO_2_	nanofibres	electrospinning	CO_2_	1000	300	3.7	24/92 s	100 ppm	[85]
La/ZnO	nanopowder	hydrothermal	CO_2_	5000	400	65	90/38 s	100 ppm	[86]
ZnO/Ca	nanopowders	modified sol-gel	CO_2_	50 000	200	∼9	—	2500 ppm	[87]
Ca/ZnO	thin film	wet chemical	CO_2_	25 000	400	∼2.5	87/132	5000 ppm	[88]
polyaniline/LaFeO_3_	microsphere	hydrothermal	CO_2_	20 000	RT	31.8	334.2/86.8 s	5000 ppm	[89]
ZnO	thin film	spray pyrolysis	CO_2_	400	350	64	75/108 s	25 ppm	[90]
ZnO/Na	nanostructured films	spin-coated	CO_2_	50	RT	81.9	283/472 s	—	[91]
Gd/CeO_2_	nano-pellets	co-precipitation	CO_2_	800	250	45	—	—	[92]
ethynylated-thiourea	solution	reaction	CO_2_	1000	RT	25	1/3 min	249 ppm	[93]
W/ZnO	nanorods	mechanochemical combustion	CO_2_	1000	450	∼65	∼15/∼20 s	100 ppm	[94]
MWCNT	nanotube	DLICVD	CO_2_	5000	30	2.1	30.2/49.6 s	1670 ppm	[95]
CuO–Cu*_x_*Fe_3−*x*_O4	nanocomposite thin film	RF sputtering	CO_2_	5000	250	0.50	9.5/— h	—	[96]
Ag–BaTiO_3_–CuO	thin films	RF sputtering	CO_2_	5000	250	0.28	15/10 min	500 ppm	[97]
La_1−*x*_Sr*_x_*FeO_3_	nanocrystalline powders	sol–gel	CO_2_	2000	380	0.25	11/15 min	500 ppm	[98]
CdO	nanowires	microwave-assisted wet chemical	CO_2_	5000	250	0.01	3.33/5 min	2000 ppm	[99]
SnO_2_/ZnO	composites	screen printing	CO_2_	60	RT	∼0.79	—	∼20 ppm	[100]
La_2_O_2_CO_3_	nanoparticles	hydrothermal	CO_2_	5000	300	0.62	53/120 s	300 ppm	[101]
TiO_2_–PANI	thin film	spin coating	CO_2_	1000	30	53	9.2/5.7 min	—	[102]
MoWO_3_	nanostructured thin films	RF magnetron co-sputtering	CO_2_	0.5	RT	∼29.2	6.53/8.05 s	—	[103]
ZnO	nanorods	hydrothermal	CO_2_	1000	150	0.9	11/30 s	100 ppm	[104]
Al_2_O_3_/MWCNT	nanotubes	gel cast	CO_2_	450	RT	0.07	53.7/— s	50 ppm	[105]
La2O_3_/SnO_2_	nanofibres	electrospinning	CO_2_	100	300	5.1	—	—	[106]
ZnO	thin film	magnetron sputtering	CO_2_	1000	300	1.01	<20/20 s	500 ppm	[107]
ZnO	nanowires	sol-gel	CO_2_	15	200	1.04	8/40 s	—	[108]
SnO_2_	nanoparticles	co-precipitation	CO_2_	2000	240	∼1.3	∼350/4 s	2000 ppm	[109]
SnO_2_	nanoparticles	mechanical milling	CO_2_	1000	400	1.1	—	—	[110]
CdO	nanopowders	co-precipitating	CO_2_	5000	250	1.03	200/300 s	500 ppm	[111]
CuO	porous film	pneumatic spray pyrolysis	CO_2_	100	RT	1.04	10/6 s	20 ppm	[112]
YPO_4_	nanobelts	surfactant-assisted colloidal	CO_2_	200	400	—	200/136 s	—	[113]
La_2_O_3_	microrods	chemical bath	CO_2_	350	250	∼1.9	∼50/73 s	100 ppm	[114]
LaOCl	nanopowders	sol-gel	CO_2_	2000	260	3.40	—	—	[115]
RGO	nanosheets	airbrushing		5000	RT	1.02	—	—	[116]
Nd_2_O_2_CO_3_	nanoparticles	sol-gel	CO_2_	1000	350	∼4	—	300 ppm	[117]
La_2_O_2_CO_3_	nanorods	co-precipitation	CO_2_	3000	325	7.08	15/30 min	100 ppm	[118]
CNT	nanotubes	CVD	CO_2_	800	RT	1.1	—	—	[119]
LaFeO_3_	nanoparticles	sol-gel	CO_2_	2000	300	∼2.2	240/480 s	—	[120]
GdCoO_3_	nanoparticles	solution polymerization	CO_2_	—	400	1.1	10/5.3 s	—	[121]
RGO	nanosheets	hydrogen plasma	CO_2_	769	RT	0.13	—/∼4 min	300 ppm	[122]
Yb_0.8_Ca_0.2_FeO_3_	nanoparticles	sol-gel	CO_2_	5000	260	2.01	24/31 s	1000 ppm	[123]
graphene	nanosheets	mechanical cleavage	CO_2_	100	RT	∼1.3	8/10 s	10 ppm	[124]
CNT	random CNT network	CVD	CO_2_	500	RT	1.2	385/412 s	100 ppm	[125]
few-layered graphene	nanosheets	electrochemical exfoliation	CO_2_	200	RT	3.8	11/14 s	3 ppm	[126]
La_0.875_Ca_0.125_FeO_3_	nanoparticles	sol-gel	CO_2_	1000	320	∼1.7	—	—	[127]
In_2_Te_3_	thin film	flash evaporation	CO_2_	1000	RT	1.1	0.05/—s	100 ppm	[128]
In_2_Te_3_	thin film	SHI irradiation	CO_2_	1000	RT	1.1	15–20/—s	—	[129]
CNT	nanotubes	CVD	CO_2_	800	RT	1.02	12/56 s	50 ppm	[130]
LaOCl–SnO_2_	porous film	electrostatic spray pyrolysis	CO2	2000	425	∼1.4	—	400 ppm	[131]
CuO–BaTiO_3_	thin film	magnetron sputtering	CO_2_	5000	300	∼1.1	>120/80 s	500 ppm	[132]
CuO–BaTiO_3_	thin film	magnetron sputtering	CO_2_	5000	RT	3.3	300/300 s	500 ppm	[133]
CuO–BaTiO_3_	thin film	magnetron sputtering	CO_2_	1000	250	∼1.8	>90/120 s	350 ppm	[134]
LaFeO_3_–SnO_2_	porous film	mixing	CO_2_	4000	250	2.7	<20/—s	—	[135]
ZnO_2_–CuO	thick film	mixing	CO_2_	4000	300	1.3	—	400 ppm	[136]
Ca–ZnO	nanoparticles	sol-gel	CO_2_	5000	450	2.1	—	—	[87]
_0.4_SnO_2_–_0.6_WO_3_	nanoparticles	mixing	CO_2_	300	RT	∼1.1	127/42 s	100 ppm	[137]
Cr–TiO_2_	thin film	magnetron sputtering	CO_2_	10 000	55	∼1.2	—	—	[138]
CuO–Cu*_x_*Fe_3−*x*_O4	thin film	RF sputtering	CO_2_	5000	250	1.9	9.5/—h	1000 ppm	[96]
BaCO_3_–Co3O_4_	nanoparticles	grounding	CO_2_	1000	150	∼1.1	192/215 s	500 ppm	[139]
ppy-FeCl_3_	porous film	chemical oxidative polymerization	CO_2_	700	RT	∼1.6	210/1560 s	100 ppm	[140]
SnO_2_-LaOCl	nanopowders	impregnation	CO_2_	2000	350	1.02	—	500 ppm	[141]
SnO_2_/ZIF-67	core–shell	mixing	CO_2_	5000	205	1.2	220/25 s	—	[142]
SnO_2_-LaOCl	nanowires	drop-coating	CO_2_	4000	400	6.8	15/19 s	250 ppm	[143]
SWCNT/PIL	nanotubes	grinding	CO_2_	10	RT	1.02	<60/—s	500 ppt	[144]
ZnO-LaOCl	nanowires	drop-coating	CO_2_	2000	400	3.5	∼15/∼17 s	—	[145]
SnO_2_-La	nanoparticles	impregnation	CO_2_	500	250	1.4	∼20/∼75 s	50 ppm	[146]
TiO_2_/Al_2_O_3_	thin films	ALD	CO_2_	25	RT	1.4	—	5 ppm	[147]
CuO–BaTiO_3_/Ag	thin film	magnetron sputtering	CO_2_	5000	300	1.2	120/80 s	500 ppm	[97]
CuO/BaTiO_3_	spheres decorated leaves	co-precipitating	CO_2_	700	120	1.2	5/18 s	∼51 ppm	[148]
RGO/PEI	thin films	airbrushing	CO_2_	3667	RT	∼1.01	14/14 s	20 ppm	[149]
ZnO/Ag–CuO	spheres decorated with leaves	impregnation	CO_2_	1000	320	1.3	76/265 s	100 ppm	[150]
In_2_O_3_/CaO	mesoporous	impregnation	CO_2_	2000	230	∼1.8	—	300 ppm	[151]
La_2_O_3_/Pd	porous film	dipping	CO_2_	500	250	1.4	105/145 s	250 ppm	[152]
La_2_O_3_/Pd	thin film	dipping	CO_2_	400	250	2.8	80/50 s	50 ppm	[24]
graphene/Sb_2_O_3_	thin film	*in situ* chemical route	CO_2_	50	RT	∼1.2	16/22 s	—	[25]
PILs/La_2_O_2_CO_3_	thin films	drop-casting	CO_2_	2400	RT	∼1.1	300/—s	150 ppm	[153]

Many researchers reported the preparation of multi-walled carbon nanotubes (MWCNTs)-based CO_2_ gas sensors using different methods such as the chemical vapour deposition technique followed by the spin coating technique and low-cost gel cast method. The MWCNTs grown on Co nanoparticles exhibited high sensitivity of 2.1 at 5000 ppm, towards CO_2_ gas, with fast response and recovery time as 30.21 s and 49.62 s, respectively, at room temperature, while CNT composite-based film sensors with different concentrations of alumina (Al_2_O_3_) show excellent changes in conductance when exposed to CO_2_ ranging from 50 to 450 ppm. The speed and stability of CNT based sensors are found to be favourable for CO_2_ sensing [95,105]. SnO_2_-based CO_2_ sensors have attracted favourable attention of the research community for the detection of different gases owing to effective enhancement in sensitivity.

**Figure 6.**
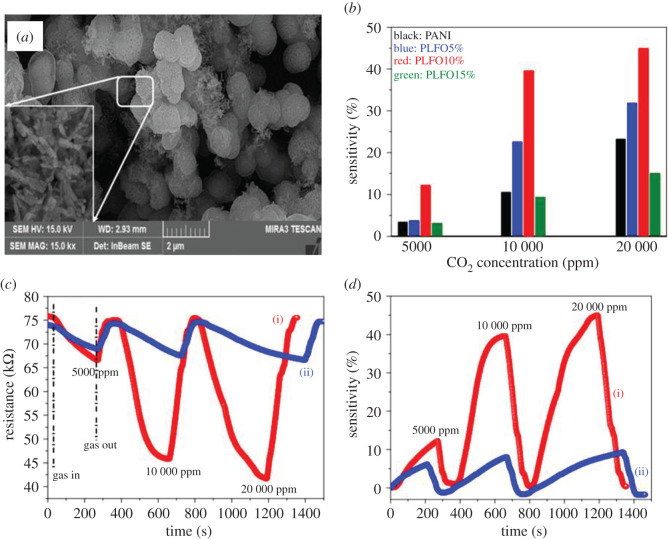
(*a*) Field emission scanning electron microscopy image, (*b*) sensitivity versus CO_2_ concentration at room temperature (27°C) and (approx. 90%) humidity of mesoporous LaFeO_3_ microspheres, (*c*) resistance shift, and (*d*) sensitivity of 10% PANI/LaFeO_3_ film sensor exposed to different concentrations of the CO_2_ gas (i) at initial test and (ii) after 1 year. Reprinted with permission from [89].

Recently, many reports have been published on Au-La_2_O_3_-doped SnO_2_ nanofibres (NFs) (figure 6*a*), porous LaFeO_3_/SnO_2_ nanocomposites and LaFeO_3_ microspheres fabricated by different chemical methods in which incorporation of metals or metal oxides enhance the response behaviour of the sensor towards CO_2_ gas. A LaFeO_3_/SnO_2_ thick film-based sensor shows low response time (<20 s) at 250°C. While a mesoporous polyaniline (PANI)/LaFeO_3_ (LFO) nanocomposite (PLFO) gas sensor with 10 wt% LFO shows 13.20 times higher gas sensitivity to 20 000 ppm of CO_2_ in comparison to a pure PANI gas sensor as shown in figure 6*b*. These sensors have long-term stability, even after 1 year the response is reasonably good as shown in figure 6*c*,*d*. The La_2_O_3_ (5 wt%)-doped SnO_2_ NFs prepared by an electro spinning process shows effective improvement in the response of many kinds of gases, especially CO_2_. In addition, Au particles (approx. 15–20 nm) sputtered on 5 wt% La_2_O_3_/SnO_2_ can improve the response of a gas sensor by 50 [89,106,135]. Copper oxide has also been an attractive material to detect CO_2_ in different nanodimensions such as thin films, nanocomposites and nanostructures. CuO–Cu*_x_*Fe_3−*x*_O_4_ nanocomposite (with 0 ≤ *x* ≤ 1) based sensor prepared by RF sputtering was investigated for CO_2_, concentrations up to 5000 ppm at different operating temperatures (130–475°C) while BaTiO_3_–CuO sputtered thin film sensor has been reported to detect CO_2_ with the addition of Ag. The resistance and capacitance changes are closely related to the changes in the work function in BaTiO_3_ and CuO material. A similar study was also reported on heterostructures of Ag–CuO/BaTiO_3_ for low-temperature CO_2_ detection by the diffuse reflectance infrared Fourier transform spectroscopy (DRIFTS) technique. This metal nanocomposite-based sensor showed significant selectivity and sensitivity towards CO_2_ at 120°C with remarkable response and recovery times (less than 10 s), high repeatability and accuracy (98%) in comparison to the pure BaTiO_3_ and CuO. The CuO films deposited by the spray pyrolysis method showed good sensitivity towards different concentrations of CO_2_ [96,112,132,150].

Table 4 summarizes the materials, synthesis methods and detection parameters of a variety of MOS-based CO_2_ sensors. Over the years, the sensitivity, selectivity, detection limit and response/recovery time have been remarkably improved with the use of a wide range of materials, synthesis methods and synthesis parameters. It can be inferred that nanostructured composite materials have better sensing performance as compared to the other materials. Numerous reports have proved that the MOS-based sensors have a great potential to be used in commercial CO_2_ gas sensors.

Various methods have also been used by different researchers for the detection of CO_2_ like gas chromatography (GC), photoacoustic spectroscopy, etc [154]. In a published report, gas phase CO_2_ in the headspace of champagne glasses was monitored through combined diode laser spectrometry and micro-GC analysis. It was also discussed in this report that an excess amount of CO_2_ can even cause a very unpleasant tingling sensation perturbing both ortho- and retronasal olfactory perception [154]. Photoacoustic spectroscopy such as quartz-enhanced photoacoustic spectroscopy (QEPAS) and cantilever-enhanced laser-PAS have been reported for CO_2_ detection [155]. A report was published by Gerald Gerlach *et al*. [156] which described the various analytical methods like spectroscopy and GC for the detection of CO_2_.

### 3.2. Metal oxide semiconductor-based methane gas sensors

CH_4_ is an odourless, colourless but highly flammable GHG which has been widely employed as reliable source of energy for domestic as well as industrial applications. It is an important constituent of natural gas and used as fuel for vehicles (CNG, LNG), heat generation and also in electricity production. It is often generated from biomass combustion, coal mines, sewage treatment, animal waste and rice production, etc. [157,158]. However, CH_4_ is known for its extremely flammable and volatile nature that sometimes causes dangerous explosions within a concentration range of 4.9–15.4% [159]. Owing to safety concerns, CH_4_ detection is essential and highly sensitive and reliable sensors are required to prevent deadly explosions. Currently, MOSs-based sensors have gained significant attention for CH_4_ sensing, the summary of various MOS-based CH_4_ gas sensors with sensor parameters such as operating temperature (°C), response/recovery time (*t*_res_/*t*_rec_) and LOD, etc. are given in table 5.

**Table 5.** Summary of various MOS-based CH_4_ gas sensors.

material	structure	synthesis method	target gas	*C* (ppm)	operating temp. (°C)	response	*t*_res_/*t*_rec_	LOD	ref.
TiO_2_	nanorods	hydrothermal	CH_4_	60	RT	6028	—	5 ppm	[160]
VO_2_	nanorods	thermal evaporation	CH_4_	500	RT	35	75/158 s	∼100 ppm	[161]
Pt/VO*_x_*	thin films	magnetron sputtering	CH_4_	500	RT	18.2	∼16.7/∼33 s	∼500 ppm	[162]
Au/VO_2_	nanosheets	CVD	CH_4_	500	RT	∼70	∼50/∼100 s	∼100 ppm	[163]
Pd/SnO_2_/rGO	nanoparticles	hydrothermal	CH_4_	4000	RT	2.07	10 min/—	—	[164]
SnO_2_	nanoparticles	sol-gel	CH_4_	20 000	80	74	16/70 s	21 126 ppm	[165]
SnO_2_/WO_3_	nanosheets	impregnation	CH_4_	5000	90	1.5	∼1.5/∼100 s	5 ppm	[166]
γ-Fe_2_O_3_	nanoparticles	green synthesis	CH_4_	100	150	∼8.5	∼10/∼40 s	1 ppm	[167]
SnO_2_	quantum dots	sonochemical	CH_4_	5000	RT	∼10	∼170/∼200 s	—	[168]
TiO*_x_*N*_y_*	nanopowders	ball milling	CH_4_	100	RT	1010	33/38 s	20 ppm	[169]
Pd/SnO_2_	nanoparticles	sol-gel	CH_4_	937	350	12.4	6/10 s	47 ppm	[170]
Pd/PdO/S-SnO_2_	nanoomposites	green recycling	CH_4_	8000	240	7.8	8/12 s	300 ppm	[171]
PANI/polymer/MWCNTs	nanoomposites	wet synthesis	CH_4_	15	60	3.4	∼1/—s	5 ppm	[172]
Cr/SnO_2_	films	spin coating	CH_4_	250	350	∼1268	∼3.9/—s	1 ppm	[173]
VO_2_	nanoparticles	vapour transport	CH_4_	500	150	652	—	50 ppm	[174]
Pd/SnO_2_	nanoporous	hydrothermal	CH_4_	3000	340	17.6	3/5 s	—	[175]
Pd/SnO_2_	hollow spheres	hydrothermal	CH_4_	250	RT	4.88	3/7 s	—	[176]
V_2_O_5_	nanoflowers	magnetron sputtering	CH_4_	500	100	∼8	206/247 s	50 ppm	[177]
V_2_O_5_	—	magnetron sputtering	CH_4_	500	RT	17	—	—	[178]
VO_2_	nanorods	PLD	CH_4_	50	50	∼3.2	—	—	[179]
VO*_x_*-MWCNT	nanotubes	CCVD	CH_4_	100	RT	∼1.5	138/234 s	60 ppm	[180]
V_2_O_5_	nanoflakes	RF sputtering	CH_4_	3000	330	2.8	∼2.5/∼5 min	50 ppm	[181]
ZnO/Zn_2_SnO_4_	microflowers	solvothermal	CH_4_	1000	250	15.36	10/30 s	400 ppm	[182]
SnO_2_/NiO	porous nanosheets	immersion-calcination	CH_4_	7000	330	15.2	28/44 s	500 ppm	[183]
Pd/SnO_2_–rGO	nanocomposites	hydrothermal	CH_4_	12 000	RT	∼9.3	∼5/7 min	800 ppm	[164]
G-C_3_N_4_/ZnO	flower-like/hierarchical	precipitation–calcination	CH_4_	1000	320	∼2.6	30/200 s	100 ppm	[184]
SnO_2_	nanorods	hydrothermal	CH_4_	10 000	150	24.9	369/350 s	1000 ppm	[185]
NiO/rGO	nanocomposite	hydrothermal	CH_4_	4000	260	15.2	16/20 s	500 ppm	[186]
ZnO/rGO	hybrid composite	hydrothermal	CH_4_	4000	190	18.5	50/60 s	100 ppm	[187]
Pt/SnO_2_	nanofibers	electrospinning	CH_4_	1.11	350	4.5	30/150 s	1 ppm	[188]
Fe/SnO_2_	thick films	simultaneous precipitation	CH_4_	1000	350	0.67	—	250 ppm	[189]
Ca/Pt/SnO_2_	thin films	ion beam sputtering	CH_4_	5000	400	17	—	5000 ppm	[190]
SnO_2_	mesopores	nanocasting	CH_4_	4000	600	0.6	∼2/—min	1000 ppm	[191]
Pd/SnO_2_	nanopores	surfactant (CTABr)	CH_4_	5000	600	20	∼10/∼20 s	∼1300 ppm	[192]
MoO_3_	paste	—	CH_4_	500	500	10	∼6/∼8 min	—	[193]
Pd–Al_2_O_3_/SnO_2_	catalytic thick film	—	CH_4_	2000	450	∼5	∼100/—ms	∼2000 ppm	[194]
WO_3_/SnO_2_	nanoflowers	impregnation	CH_4_	500	110	∼2.9	—	38 ppb	[195]
SnO_2_	nanosheet	aqueous solution	CH_4_	500	RT	1.3	18/28 s	—	[196]
ZnO/NiO	porous nanosheets	hydrothermal	CH_4_	1000	340	34.2	7/33 s	300 ppm	[197]
Pt/SnO_2_	nanocomposites	hydrothermal	CH_4_	500	120	1.26	∼4.5/—min	10 ppm	[198]
Al/NiO	thin films	RF sputtering	CH_4_	100	RT	58	1373/95 s	—	[199]
Pd-sensitized ZnO	thin films	ionic layer adsorption reaction	CH_4_	2000	RT	2.46	—	667 ppm	[200]
Pd/ZnO	nanosheets	hydrothermal	CH_4_	5000	200	19.2	∼4/∼6 min	100 ppm	[201]
ZnO	thin film	electrochemical	CH_4_	100	220	∼4.8	24/72 s	—	[202]
ZnO	nanowalls	thermal evaporation	CH_4_	100	300	2	6/21 s	100 ppm	[203]
Co/ZnO	microstructure	solvothermal	CH_4_	375	140	1.05	25.2/6.6 s	100 ppm	[204]
ZnO/Pd-Ag	nanocrystalline	sol–gel	CH_4_	10 000	550	31	∼16.3/— s	200 ppm	[205]
Co/ZnO	microstructure	hydrothermal	CH_4_	100	140	3.55	19/27 s	50 ppb	[206]
ZnO–Ag	ceramics	ceramic technology	CH_4_	—	250	∼3	∼40/∼60 s	—	[207]
ZnO/Pd	nanocomposite	chemical	CH_4_	10 000	80	36.8	7/5 min	100 ppm	[208]
Fe_3_BO_6_	nanoplates	glass liquid	CH_4_	1000	252	33	1.2/2.6 min	50 ppm	[209]
RGO/ZnO	nanoparticles chain-like	anodization and thermal annealing	CH_4_	500	450	30	—	5 ppm	[210]
ZnO	microwire	carbothermal reduction	CH_4_	2000	400	26	∼12/∼28 s	200 ppm	[211]
ZnO/SnO_2_	film/nanorods	PECVD	CH_4_	100	550	∼5	2/42 s	50 ppm	[212]
Pd/Al_2_O_3_	particles	colloid mixing impregnation	CH_4_	1000	400	—	∼15/∼35 s	1 ppm	[213]
Ag/Ag_2_O–SnO_2_	nanocomposites	impregnation	CH_4_	2000	170	40	∼5/93 s	1 ppm	[214]
Fe_3_O_4_/hydrogel/MWCNTs	nanocomposites	wet synthesis	CH_4_	20	RT	—	120/— s	5 ppm	[215]
CdTiO_3_	thin films	magnetron co-sputtering	CH_4_	500	250	3.4	∼38/∼70 s	100 ppm	[216]

**Figure 7.**
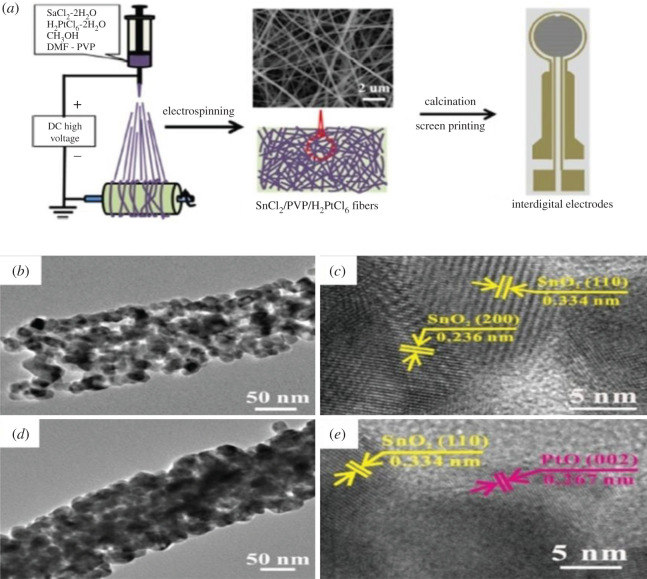
(*a*) Schematic diagram showing the process of fabrication of the gas sensor device. (*b*) TEM images of unloaded SnO_2_, (*c*) HR-TEM images of unloaded SnO_2_ (*d*) TEM images of 20 mol% Pt-loaded SnO_2_ NFs, and (*e*) HR-TEM images 20 mol% Pt-loaded SnO_2_ NFs. Reprinted with permission from [188].

Pt-loaded SnO_2_ NFs with different Pt concentration (10–30 mol%) were prepared using the electrospinning technique followed by calcinations and screen printing [188]. The sensors fabricated with 20 mol% Pt-SnO_2_ NFs (100–150 nm) show an excellent response time of 4.48 s towards 1000 ppm CH_4_ at 350°C. Figure 7*a* shows the schematic diagram of a gas sensor fabrication process, figure 7*b*,*d* are transmission electron microscopy (TEM) images, and figure 7*c*,*e* are high resolution (HR)-TEM images, that represent the nanoscale microstructure of unloaded SnO_2_ and Pt-loaded SnO_2_ NFs, respectively. The HR-TEM image (figure 7*e*), shows the simultaneous presence of SnO_2_ and PtO.

**Figure 8.**
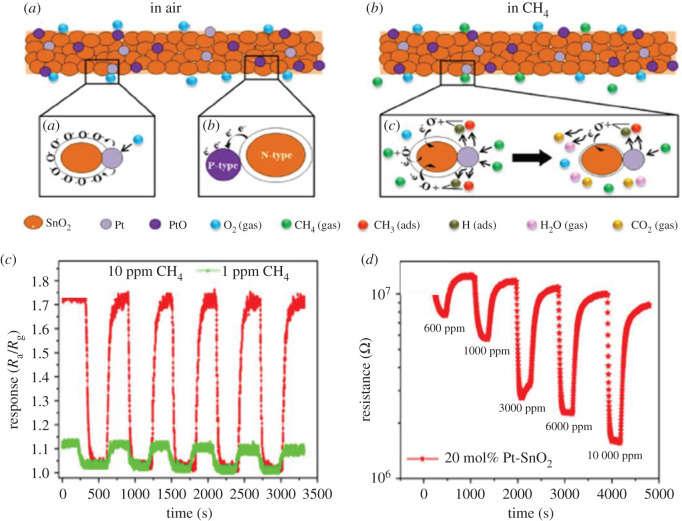
Schematic diagram of sensing mechanism of Pt-loaded SnO_2_ NFs CH_4_ for: (*a*) air and (*b*) for CH_4_, (*c*) response of 20 mol% Pt-loaded SnO_2_ NFs to 1 ppm and 10 ppm CH_4_ at 350°C, and (*d*) resistance shift for 20 mol% Pt-loaded SnO_2_ sensing property towards CH_4_ at 100°C. Reprinted with permission from [188].

Figure 8*a*,*b* presents the sensing mechanism of Pt-loaded SnO_2_ NFs for air and CH_4_, respectively, while figure 8*c* shows the sensing response of 20 mol% Pt-SnO_2_ NFs to 1 ppm–10 ppm CH_4_ at 350°C which was 2 times less than the minimum detection limit of previously fabricated SnO_2_-based CH_4_ gas sensors and (*d*) shows resistance shift for 20 mol% Pt-loaded SnO_2_ sensing characteristics towards CH_4_ at 100°C.

Cr-doped SnO_2_ nanoparticles with Cr (0–2 wt%) were synthesized by a flame spray technique and sensing film was fabricated using a spin coating technique [173]. The response time of 3.9 s at 350°C towards 1 vol% CH_4_ offered by 0.5% Cr-doped SnO_2_ sensor proves that Cr–SnO_2_ is a promising candidate for selective CH_4_ detection [173]. Pd-decorated ZnO/rGO hybrid-based CH_4_ gas sensor shows a fast sensing response time of 74 s at room temperature towards CH_4_ (25 ppm). This opens a new route to design of ternary hybrid-based CH_4_ gas sensors [217].

Titanium oxynitride (TiO*_x_*N*_y_*) nanostructures prepared by milling showed high sensitivity of 50.12% at room temperature for 20 ppm of CH_4_. The γ-Fe_2_O_3_ nanoparticles synthesized by a green approach from leaf extract provides a higher response and selectivity with short response time for CH_4_ sensing than euphorbia extracted γ-Fe_2_O_3_ nanoparticles [167]. In RGO-SnO_2_ heterostructure composite d-glucose and l-ascorbic acid reduced GO showed better CH_4_ sensing performance at room temperature than sodium borohydrate and hydrazine hydrate reduced GO, while l-ascorbic acid RGO-SnO_2_ heterostructure showed the highest CH_4_ sensing response owing to the synergistic effect between dehydroascorbic acid and surface of SnO_2_ [218]. Thermally evaporated vanadium dioxide (VO_2_) nanorods showed good sensing response with different concentrations (100–500 ppm) of CH_4_ at room temperature. The nanorod structure provides a large surface area and sensing sites for CH_4_ detection [161]. Au-decorated VO_2_ nanosheets prepared by the CVD method followed by ion sputtering provide good sensing response towards 100–500 ppm of CH_4_ at room temperature. This is attributed to the formation of a heterojunction at the interface and the creation of a depletion layer within the interface of the Au nanoparticles and VO_2_ nanosheets [163].

Magnetron-sputtered Pt-loaded VO*_x_* thin films exhibit higher response of 18.2 with 500 ppm concentration of CH_4_ at room temperature [162]. TiO_2_ nanorods-based sensors showed excellent response and recovery time of 45 s and 33 s, respectively, with high selectivity towards 199 ppm of CH_4_ owing to high surface area and point defects of TiO_2_ [160]. SnO_2_-loaded WO_3_ nanosheets showed 1.4 times higher response towards CH_4_ than pure WO_3_ at working temperature of 90°C. The highly reactive sites as a result of defect formation at the SnO_2_-loaded WO_3_ heterojunction, and oxygen chemisorptions at the dangling bonds of W atoms of WO_3_ nanosheets result in significant enhancement in the sensing behaviour [166]. The V_2_O_5_ nanostructures-based sensor showed a response of about 6.52–8% at 150°C towards 50–500 ppm CH_4_ concentration and exhibited its specificity towards the C–H bond (CH_4_) [174,177].

The ZnO–reduced graphene oxide (rGO) nanohybrid composite provides a superior sensing response of 4.52% for sensing CH_4_ at optimal temperature of 190°C than pure ZnO and rGO. The sensing mechanism is explained on the basis of the formation of a heterojunction of ZnO and rGO [183]. Vanadium oxide filled MWCNTs were developed with good response/recovery time of 16 s and 120 s, respectively, at room temperature for CH_4_ gas. The filling of vanadium oxide in MWCNTs increased the density of states around the Fermi level and adsorption sites [180]. Co-doped ZnO prepared by the hydrothermal process followed by calcinations at 600°C demonstrated superior response time and recovery time of 19 s and 27 s, respectively, towards 100 ppm concentration of CH_4_, i.e. twice that of the pure ZnO sensor [206]. SnO_2_ nanorods-nanoporous graphene hybrids with 0.05 weight ratios showed a high sensing response of 24.9–51% at 150°C towards 1000–10 000 ppm of CH_4_. This was higher than pure SnO_2_ nanorods owing to the larger surface area of the graphene hybrid and synergistic interaction between nanoporous graphene and SnO_2_ nanorods [185]. The much better sensitivity (9.5%) for 120 00 ppm CH_4_ was achieved by a Pd-doped SnO_2_/rGO composites-based sensor than Pd-doped SnO_2_/PrGO owing to catalytic action of Pd [164]. Pulsed DC-sputtered nanostructured VO_2_ thin films showed a reversible semiconductor to metal transition at 60–70°C. The film possesses sensing characteristics at a low temperature of 50°C towards 50 ppm of CH_4_ [179]. Mesoporous SnO_2_ of pore size 4.4 nm with large specific surface areas of 80 m^2^ g^−1^ were prepared by nanocasting mesoporous KIT-silica and it is found to be a highly promising sensing material for CH_4_ detection [191]. The Pd-modified ZnO nanosheets-based sensor exhibited a remarkable sensing response of 19.20 for CH_4_ (5000 ppm) with high selectivity and repeatability at 200°C than pure ZnO owing to the spill-over effect and nano-schottky barrier formation [201]. Pd-loaded mesoporous SnO_2_ hollow spheres based sensors showed response and recovery time of 3 s and 7 s, respectively, with excellent stability of 15 weeks for 250 ppm CH_4_ compared to the response shown by SnO_2_ hollow spheres [176]. The NiO nanoparticles decorated in ZnO porous nanosheets increased the specific surface area and interfacial interaction of NiO and ZnO by p–n junction formation. The NiO-decorated ZnO nanosheets exhibited excellent CH_4_ sensing performance, long-term stability, high response as compared to that of pure ZnO porous nanosheets at 340°C [197].

The research outcomes from various reports in the literature on MOS-based CH_4_ gas sensors have been summarized in table 5. Nanostructures with specific morphology such as nanorods, nanoflakes, nanofibres and nanosheets, etc., are known to exhibit better sensitivity, selectivity and response as compared to the conventional materials which is attributed to the larger surface area exhibited by them. Catalytic layers such as Pd or Au significantly improve the sensing properties of the oxide materials. Novel materials such as MWCNTs, reduced graphene oxide and their composites with metal oxides show remarkable sensing behaviour towards CH_4_.

In addition to MOS sensors other sensors are also available for CH_4_ detection such as optical sensors [219], calorimetric sensors [220], pyroelectric sensors [221], electrochemical [222] and photoacoustic detection [223]. Li *et al*. [224] reported CH_4_ detection based on QEPAS using a high-power continuous wave, single-mode diode laser with an emission wavelength at 2.3 µm and demonstrated the minimum detection limit of a CH_4_-QEPAS sensor is 7.9 ppm. A compact and portable photoacoustic gas sensor was developed for CH4 detection at 1.6 µm by a software-based wavelength stabilization scheme and the CH4 sensor achieved a minimum detection limit of 11.5 ppm at 10 s response time in the concentration range of 400–6300 ppm [225]. Zheng *et al*. [226] developed a mid-IR methane sensor using a continuous-wave inter-band cascade laser and showed that the sensor functions normally at 1.0–2.1 ppm as the pressure changed from 25 to 800 Torr. Park *et al*. [227] fabricated a calorimetric sensor with a dual-catalyst structure and successfully detected CH_4_ between 200 and 2000 ppm at temperatures of 100–400°C.

### 3.3. Metal oxide semiconductor-based nitrous oxide gas sensors

N_2_O also known as laughing gas is one of the most important GHGs which causes O_3_ layer depletion and contributes to global warming and climate change. N_2_O is also used in medical practice as an anaesthetic. It is a colourless gas having a sweet odour and produced from the breakdown of nitrogen-based fertilizers while naturally produced from oceans [22]. There are several other anthropogenic N_2_O sources such as wastewater treatment, fossil fuel combustion and industrial nylon production. It causes about 300 times more atmosphere warming per unit weight than CO_2_ and gives rise to the greenhouse effect [27]. After reaching the upper atmosphere, N_2_O molecules can stay there for 100 years. It is one of the reasons of O_3_ layer depletion, therefore, it is necessary to detect it and decompose it into N_2_ and O_2_ before release. Thus, there is huge need for the development of N_2_O gas sensors in order to protect the environment and human health. Although, there are very few reports are available on the detection of N_2_O using MOS-based sensors, some studies are listed in table 6.

**Table 6.** Summary of various MOS-based N_2_Ogas sensors.

material	structure	synthesis method	target gas	*C* (ppm)	operating temp. (°C)	response	*t*_res_/*t*_rec_ (s)	LOD	ref.
Mg_0.5_Zn_0.5_Fe_2_O_4_	nanopowder	wet chemical route	N_2_O	1600	300	19%	—	—	[228]
Au/SnO*_x_*	thin films	chemical vapour deposition (CVD)	N_2_O	100	210	11.5	—	—	[229]
SnO_2_	thick films	screen printing	N_2_O	100	RT	0.58	—	—	[230]
WO_3_	powder	co-precipitation	N_2_O	300	450	1.32	—	—	[231]
SnO_2_	powder	co-precipitation	N_2_O	300	450	1.66	—	—	[231]
ZnO	powder	co-precipitation	N_2_O	300	450	1.21	—	—	[231]
(Sr, Ca, Ba, Bi, Sm) loaded SnO_2_	powder	co-precipitation	N_2_O	300	500	4.3	—	—	[231]
Sm_2_O_3_/SnO_2_	powder	electrochemical method	N_2_O	100	450	—	90/18	35	[232]
In_2_O_3_	nanowires	anodic alumina membrane (AAM)	N_2_O	10	150	60	20/20 s	—	[233]
WO_3_	nanowire	solvothermal method	N_2_O	10	250	25	10/60 s	—	[233]
WO_3_	mat-like networked nanowire	HF-CVD	N_2_O	1	723 K	—	75 s/6 min	100 ppb	[234]

**Figure 9.**
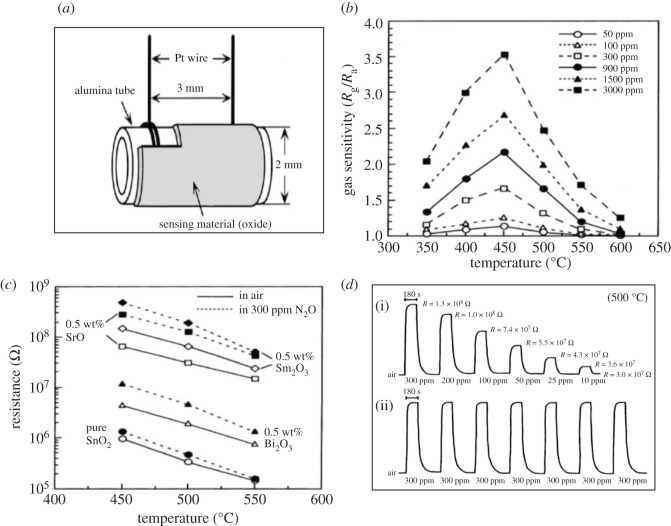
(*a*) Schematic diagram of N_2_O sensor element, (*b*) dependence of sensitivity on temperature at 50–300 ppm for pure SnO_2_, (*c*) dependence of resistance on temperature for loaded SnO_2_, and (*d*) (i and ii) response transients to N_2_O at 500 C for (0.5 wt%) SrO-loaded SnO_2_.Reprinted with permission from [231].

Au-loaded tin oxide thin films grown by ArF excimer laser-induced (MOCVD) exhibited good response of 11.5 for 100 ppm N_2_O at 210°C [229]. The (0.5 wt%) Sm_2_O_3_ loaded SnO_2_ exhibited high sensitivity about 1.5 times higher than pure SnO_2_ towards N_2_O at 475°C and allowed the detection of 35 ppm N_2_O in air [232]. Kanazawa *et al*. [231], reported SnO_2_-loaded with 0.5 wt% SrO showed 3 times higher response than unloaded SnO_2_ towards N_2_O of 10–300 ppm at 500°C. A schematic of an SnO_2_-based sensor is shown in figure 9*a*. The sensitivity versus temperature with various concentrations of N_2_O is shown in figure 9*b–d*. It has been found that (0.5 wt%) SrO-loaded SnO_2_ is found to be more sensitive towards N_2_O than pure SnO_2_. Rout *et al*. [233] investigated the sensing characteristics of ZnO, In_2_O_3_ and WO_3_ nanowires for N_2_O detection and found that In_2_O_3_ nanowires of approximately 20 nm exhibited sensitivity of 60 for 10 ppm having response and recovery time of about 20 s at 150°C. Also, the WO_3_ nanowires prepared by the solvothermal method showed sensitivity of about 25 for 10 ppm having response and recovery time of 10 s and 60 s, respectively, at 250°C. The sensitivity of In_2_O_3_ and WO_3_ nanowires are unaffected up to 90% of relative humidity. Deb *et al*. [234] reported the WO_3_ nanowire mats and nanoparticle films were deposited by the hot filament chemical vapour deposition (HF-CVD) method. The response of tungsten trioxide nanowire (mat-like, nanowire networks) and nanoparticle thin films was in the temperature range of 373–773 K. The mat-like nanowire network exhibited higher resistivity change response in comparison to nanoparticle films at temperatures above 523 K. The nanowire mats showed the high sensitivity with much improved response and recovery times of 75 s and 6 min, respectively, at 723 K. The calculated activation energy from the time constant–temperature plot was about 26 kcal mol^−1^ for the nanowire device.

However, there are several other methods available for the detection of N_2_O such as chromatography [235], optical methods [236], laser absorption spectroscopy [237], photoacoustic spectroscopy [238] and amperometric microsensor [239]. Ryu *et al*. [240] used a GS-electron capture detector (GC-ECD) for the determination for N_2_O of different concentrations in ancient-air-trapped ice cores. The quantum cascade laser absorption spectrometer, called ‘QCLAS’ was developed by Mappe *et al*. [241] in order to monitor *in situ* GHGs like N_2_O and CH_4_ at high temporal resolution with a high accuracy. Kang *et al*. [242] evaluated the repeatability of photoacoustic spectroscopy and found it to be 1.12%, which is less than the repeatability of 3.0% according to the International Organization for Standardization (ISO) 1564 standard. The detection limit was obtained at 0.025 ppm and the response time was found to be 3 min and 26 s. The major advantage of photoacoustic detection is that there is no need of any complex instrumentation to work at atmospheric pressure. GC-ECD is the most widely employed analytical method for measuring N_2_O. It is a low-cost method as compared to other analytical techniques [243]. However, optical techniques such as Fourier-transform infrared spectroscopy (FTIR) and laser absorption spectroscopy have major advantages over chromatographic techniques as they are capable to carry out continuous measurements. Laser absorption spectroscopy allows rapid as well as highly sensitive measurements and have lower interference of other trace gases but it is an expensive technique and also cryogenic cooling is a major requirement of this technique [244].

Electrochemical amperometric sensors have the main advantages of gas detection because of their good sensitivity, simplicity, ease of use and low cost of device construction [245]. Some new methods like amperometric biosensors [239] and optical fibres [246] are still developing for N_2_O detection. There are a number of analytical techniques other than MOS-based sensors for the detection of N_2_O and each technique has its own advantage and disadvantage. From past years it is possible to measure very low concentrations of N_2_O very fast. The major requirement in N_2_O detection is a cheap, accurate and easily deployable sensor which can collect data over extensive areas.

Thus, the outcomes from above-mentioned reports on MOS-based N_2_O gas sensors suggests that different morphologies such as nanowire, nanopowder, mat-like networked nanowire exhibited better sensitivity and response as compared to conventional materials owing to a large surface area acquired by them. Additionally, SnO_2_ and WO_3_ materials have been studied widely for the N_2_O detection while In_2_O_3_ also shows remarkable sensing behaviour towards N_2_O.

### 3.4. Metal oxide semiconductor-based ozone gas sensors

The peak concentrations of O_3_ are now substantially higher than the preindustrial levels, owing to many sources of pollution [247,248]. In the Earth's lower (near ground) atmosphere, O_3_ is formed when pollutants emitted by cars, power plants, industrial boilers, refineries, chemical plants and other sources react chemically in the presence of sunlight. O_3_ pollution naturally appears as a concern during the summer, when the weather conditions form ground-level O_3_ owing to the high temperatures. O_3_ acts as a GHG, absorbing some of the IR energy radiated by the Earth's surface. Measuring the quantity of the GHG potency of O_3_ is not easy because its concentration is not uniform across the globe. However, the most widely accepted scientific analysis relating to climate change/global warming suggests that the radiation absorption by atmospheric O_3_ is about 25% that of CO_2_ [249]. The environmental O_3_ does not show any strong global effects owing to its short life but O_3_ in the atmosphere has a radiative forcing effect approximately 1000 times as strong as CO_2_ [250].

The demand for O_3_ sensors is increasing gradually for monitoring and controlling gas emission in the atmosphere. The MOS-based gas sensing device has drawn the interest of many researchers to improve the sensing performance in terms of its selectivity, sensitivity, operating temperature, response and recovery time [251–255]. Various NMOS-based O_3_ gas sensors, such as ZnO [255–263], SnO_2_ [264–266], WO_3_ [267–269], CuO [270], In_2_O_3_ [271–276] and ZnO/SnO_2_ [277], etc., with their fabrication method and sensor parameters such as operating temperature (°C), selectivity, stability, response/recovery time (*t*_res_/*t*_rec_) and LOD have been demonstrated in table 7. The sensing mechanism of NMOS for O_3_ is based on the adsorption of gaseous molecules (oxygen, of air) on the surface of the metal oxide. This oxygen (O_2_) molecule becomes partially ionized with the core electrons to form oxygen species on the surface of the metal oxide: O_2s_^−^, O_s_^−^ or O_s_^2−^ [290,291]. When O_3_ is introduced, O_3_ molecules react with oxygen species on the surface of sensing materials, forming oxygen gas (O_2_), in addition to releasing the trapped electron back to the core. During this process, the resistance of sensing materials is increased with an increase in O_3_ gas concentration, based on the following reactions [280,292]:

3.1 $${\left(O_{2}\right)}_{\mathrm{gas}}+{\left(O_{2}\right)}_{\mathrm{ads}},$$

3.2 $${\left(O_{2}\right)}_{\mathrm{ads}}+e^{-}{\left(O_{2}^{-}\right)}_{s}$$

3.3 $${\left(O_{2}^{-}\right)}_{s}+e^{-}{\left(2O^{-}\right)}_{s}$$

3.4 $${\left(O^{-}\right)}_{s}+{\left(O_{3}\right)}_{\mathrm{gas}}{\left(2O_{2}\right)}_{\mathrm{gas}}+e^{-}.$$

**Table 7.** Summary of various MOS-based O_3_ gas sensors.

material	structure	synthesis method	target gas	*C* (ppm)	operating temp. (°C)	response	*t*_res_/*t*_rec_	LOD	ref.
ZnO	nanorods	hydrothermal	O_3_	0.1	250	∼3	14/60 s	0.06 ppm	[256]
ZnO	urchin-like nanorods	CVD	O_3_	280 ppb	200	∼100	—	280 ppb	[257]
ZnO	nanostructures	aqueous chemical	O_3_	1	RT	∼3.9	∼3/∼5 min	∼1 ppm	[258]
ZnO	nanosheets	hydrothermal	O_3_	100 ppb	300	90.5	—	—	[259]
ZnO	powders	polymeric precursor	O_3_	80 ppb	250	5.0	11/14 s	33 ppb	[255]
ZnO	thin films	RF magnetron sputtering	O_3_	49.9	RT	15	∼10/∼30 min	0.32 ppm	[260]
ZnO	nanowire	ALD	O_3_	600 ppb	25	1.2	—	100 ppb	[261]
ZnO	nanostructures, flower-like shape	microwave-assisted hydrothermal	O_3_	100 ppb	120	∼12	9.6/45.6 s	—	[262]
ZnO	films	spray pyrolysis	O_3_	182 ppb	RT	23	1/10 min	16 ppb	[263]
SnO_2_	thin films	spray pyrolysis	O_3_	∼1	200	4	5/2 s	100 ppb	[264]
SnO_2_	thin films	SILD	O_3_	∼1	200	∼100	4/100 s	1000 ppb	[265]
SnO_2_	thin films	sol–gel	O_3_	0.5	RT	3.1	15/12 min	—	[266]
SnO_2_	thin films	sol–gel	O_3_	217 ppb	RT	∼1.2	2/3 min	58 ppb	[278]
WO_3_	thin films	—	O_3_	68 ppb	500	∼360	∼300/—s	13 ppb	[267]
WO_3_	thin films	RF sputtering	O_3_	80 ppb	400	5	—	10 ppb	[268]
WO_3_	thin films	RF-magnetron sputtering	O_3_	0.8	250	16	1/<60 s	0.03 ppm	[269]
CuO	thin films	RF sputtering	O_3_	500 ppb	250	—	∼1/∼15 min	—	[270]
PdO	thin films	thermal sublimation	O_3_	100 ppb	175	∼1700	—	10 ppb	[279]
In_2_O_3_	thin films	spray pyrolysis	O_3_	∼1	250	∼100	∼10/180 s	1000 ppb	[271]
In_2_O_3_	thin films	sol-gel	O_3_	400 ppb	100	20	—	200 ppb	[272]
In_2_O_3_	nanoparticles	MOCVD	O_3_	60 ppb	RT (UV)	∼4	—	10 ppb	[273]
In_2_O_3_	nanoporous particles	nanocasting	O_3_	0.22	RT, UV assisted	200	2.5/5.3 min	50 ppb	[274]
In_2_O_3_	urchin-like microspheres	solvothermal	O_3_	40 ppb	150	21.5	60/40 s	10 ppb	[275]
In_2_O_3_	nanoparticles	hydrothermal	O_3_	60 ppb	RT with UV	∼1.62	—	10 ppb	[276]
Au/TiO_2_	core–shells	sol–gel	O_3_	0.5	RT	1.15	2/5 s	0.4 ppm	[280]
Co/SnO_2_	thin films	spray pyrolysis	O_3_	1	270	∼10	—	1000 ppb	[281]
IN2O/SiN*_x_*	films	RF sputtering and PECVD	O_3_	40 ppb	195	∼171	3/7.5 min	20 ppb	[282]
SnO_2_/SWNTs	thin films	sol–gel	O_3_	1 ppb	RT	∼0.88	—	20 ppb	[283]
WO_3_/rGO	nanocomposites	liquid flame spray	O_3_	10 ppm	150	∼370	17.1/32.7 s	0.5 ppm	[284]
ZnO/SnO_2_	heterojunctions	hydrothermal	O_3_	0.3	RT	37.5	13/90 s	20 ppb	[277]
ZnCo_2_O_4_	microspheres	co-precipitation	O_3_	560 ppb	200	0.23	8/10 s	80 ppb	[285]
Pt/TiO_2_–SnO_2_	nanomaterial	dip coating	O_3_	2.5	RT (UV)	∼250	160/50 s	500 ppb	[286]
Zn_0.95_Co_0.05_O	thin film	spray pyrolysis	O_3_	1040 ppb	250	0.4	46/62 s	20 ppb	[287]
Zn_0.95_Co_0.05_O	thin films	polymeric precursors	O_3_	0.89	200	∼3	46/360 s	42 ppb	[288]
SrTi_0.85_Fe_0.15_O_3_	thin films	electron beam deposition	O_3_	0.8	260	∼3	26/72 s	0.1 ppm	[251]
SrTi_1−*x*_Fe*_x_*O_3_	thin films	polymeric precursor	O_3_	600 ppb	250	170–580	∼2/<5 min	75 ppb	[289]

It is well known that the gas sensing mechanism of O_3_ involves the adsorption of O_3_ gas molecules onto the surface of sensing materials. This creates an electron-depletion layer which is owing to the adsorption of ions. Thus, this electron-depletion layer increases the potential barrier and consequently increases in resistance of sensing materials [259]. One of the remarkable features of NMOS-based O_3_ gas sensors is their fast response owing to the high reduction rate of O_3_ molecules.

A spray pyrolysis deposited SnO_2_ thin film sensor exhibited a very fast response time of 5–10 s towards 4–1 ppm O_3_ at 200°C [264], while SnO_2_-based thin sensitive double layers (approx. 100 nm) demonstrated a response value (approx. 1.2) and response/recovery times (2/3 min) towards 217 ppm O_3_ at room temperature with LOD of 58 ppm.

One-dimensional ZnO nanorod-like structures prepared by the hydrothermal method showed response/recovery times of 14/60 s towards 0.1 ppm O_3_ with long-term stability (six months) at 250°C [256]. A high-density porous zinc oxide (ZnO) nanosheets (NSs)-based microelectromechanical systems (MEMS) gas sensor was designed for O_3_ detection [259]. The sensor demonstrated high response (90.5) towards 100 ppb O_3_ at an operating temperature of 300°C with long-term stability and repeatability, as it was tested for 168 h. In addition, a ZnO nanosheets-based sensor provides excellent selectivity towards O_3_ compared to 100 ppb carbon monoxide, methanol, acetone, ethanol as shown [259]. The sensing response of the porous ZnO NSs is attributed to the change in concentration of electrons in the conduction band upon O_3_ gas exposure, as a result conduction band or acceptor level holes move to the high-energy surface sites residing in the band gap [259].

ZnO multi-wires (50 nm) prepared by the microwave-assisted hydrothermal method provided fast response/recovery times of 9.6/45.6 s with a very high sensitivity to 100 ppb O_3_ gas at an operating temperature of 120°C [262].

Other NMOSs such as WO_3_, CuO and PdO have also been reported as good sensing materials for O_3_ gas sensing [269]. RF magnetron-sputtered WO_3_ nanostructured thin film exhibited fast response/recovery time value of 1/<60 s with a low LOD (30 ppb) at a temperature of 523 K [269] while sputtered deposited CuO thin film showed a response time of 60 s and very long recovery time of 15 min for 500 ppb O_3_ at 250°C [270].

PdO ultrathin films of thickness (5–10 nm) were grown on SiO_2_/Si substrate by the thermal oxidation process. The PdO ultrathin film sensor provided a very high response value of approximately 1700 towards 100 ppm O_3_ with superior low LOD (10 ppm), high signal stability and reproducibility at a moderate temperature of 175°C [279].

A nanostructured In_2_O_3_ thin film-based gas sensor prepared by sol-gel processes was found to be another suitable material for O_3_ sensing. The sensor showed high sensitivity with a response of 20 for 400 ppm O_3_, good reproducibility and selectivity against the other interfering gases at 100°C [272]. A photon stimulated ozone sensor based on In_2_O_3_ nanoparticles (7 nm) was fabricated by the MOCVD technique and the sensor was observed to be highly sensitive and stable in highly humid conditions for 10 ppm O_3_ at room temperature [273].

The In_2_O_3_ microsphere (15 nm) based sensor has also been found very impressive with its fast response/recovery time of 60/40 s for 40 ppm O_3_ at 150°C operating temperature. The sensor showed good selectivity, stability, reproducibility and linear response towards O_3_ (40–240 ppm) with extremely low LOD of 10 ppm [275].

Nanostructured O_3_ gas sensors decorated by noble metals Au, Cu, etc. [280,281] and transition metals Co, Fe, etc. [281] have become one of the effective approaches to improve gas sensing performance of O_3_. In case of O_3_, the selectivity i.e. detecting and quantifying pure O_3_ from the mixture of various gases in the atmosphere at room temperature is a difficult task and NMOSs surfaces modified by noble metals have proved to be of great use to address this problem. An Au-decorated TiO_2_ core–shell nanoparticle (9–12 nm) prepared by using the sol-gel method showed super-fast response/recovery times of 2/5 s towards (0.5–7.0) ppm O_3_ at room temperature. This sensor exhibits excellent selectivity towards O_3_ against the various gases such as CH_3_OH, C_2_H_5_OH, H_2_ and NO_2_ [280]. The co-doped SnO_2_ thin films sensors were grown on quartz substrates by the spray pyrolysis method and it was found that 4 wt% of cobalt nanoparticles enhanced the gas sensing response (approx. 10 for 1 ppm O_3_) at 270°C [281].

The gas sensing performance of the NMOSs-based gas sensor can also be enhanced by amalgamating them with other metal oxides or carbon-based nanostructures [282]. For these MOS nanocomposites, the heterojunctions (the interfaces between different metal oxides) can accelerate the response speed of the sensor via effectively enhancing the electron transfer between different species. Besides the composite oxides, many mesoporous structures have been developed to improve the sensing parameters especially the sensitivity and response speed of these composite oxides-based sensors. The accumulation of nanoparticles to the composite oxides are beneficial for the adsorption and desorption of gas molecules, thus most sensors composed of the composite oxides exhibit very fast response times at room temperature [282].

The In_2_O/SiN*_x_* thin film was grown on YX LiTaO_3_ substrates using a sputtering technique and a SAW-based sensor was designed [282]. The sensor showed a large response (approx. 171) towards 40 ppm O_3_ at 195°C. The sensor was found to perform very well as far as reversibility and repeatability were considered but long response/recovery time of 3/7.5 min made it unfit for practical application. The ZnO/SnO_2_-based composite heterojunctions were prepared by using a hydrothermal method [277]. The composite heterojunction is composed of a ZnO needle with (100) orientation of the hexagonal phase of ZnO and SnO_2_ nanoparticles with the rutile phase of SnO_2_. The sensor displayed an extremely fast response/recovery time of 13/90 s for 0.3 ppm O_3_ at room temperature, after UV exposure.

In addition, this sensor has very low LOD (0.3 ppm of O_3_ with excellent sensing response, 37.5) and cross sensitivity/selectivity against the other interfering gases such as NH_3_ (1 ppm), NO_2_ (1 ppm) and CO (1 ppm).

The nanocomposites combining WO_3_ nanoparticles (220 nm) and rGO nanosheets were fabricated on the surface of graphene nanosheets by a liquid flame spray method using WCl_6_ and rGO as a precursor to enhance the gas sensing performance for O_3_. This nanocomposite-based sensor exhibited very large response (approx. 370) towards O_3_ as compared to pure rGO or WO_3_ nanoparticles. The sensor showed the good response/recovery time of 17.1/32.7 s (for 0.3 wt% rGO) for 10 ppm O_3_ at 150°C and also very low (0.5 ppm) LOD for the O_3_ gas [284]. A single-walled carbon nanotubes/SnO_2_-based O_3_ gas sensor exhibited a fast response/recovery time with good sensitivity compared to a pure SnO_2_-based sensor at room temperature. The CNT/SnO_2_-based sensor has good LOD (1 ppm) of O_3_ and remarkable stability (over one month) [283].

The gas sensing performance of O_3_ gas sensors can also be improved by doping with metal ions in NMOSs owing to the increased number of active sites and defects on the surface of NMOS nanocrystals. These active sites enhance the amount of oxygen species and increase the adsorption of gas molecules on the sensor's surface. For the improvement in gas sensing performance of the NMOS, doping of metal ions such as Zn^2+^, [285], Co^3+^, [287,288] and Fe^3+^, [251,289] has been reported in the literature.

**Figure 10.**
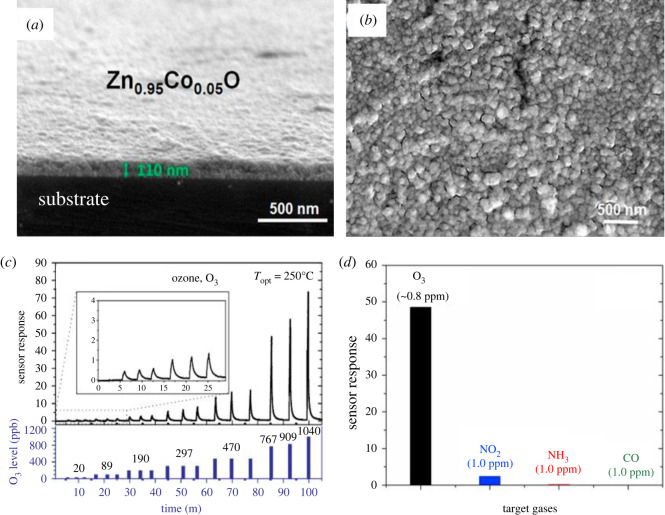
(*a*) SEM cross-sectional image, (*b*) surface views of Zn_0.95_Co_0.05_O thin film, (*c*) ozone (O_3_) sensor response of Zn_0.95_Co_0.05_O thin film at 250°C exposed to different O_3_ levels (20–1040 ppb) (inset shows a detailed region of sensor response towards 20 and 89 ppb of O_3_ gas), and (*d*) comparison of the sensor responses of the Zn_0.95_Co_0.05_O thin film exposed towards different gases at an operating temperature of 250°C. Reprinted with permission from [287].

The Zn_0.95_Co_0.05_O thin film prepared by the spray pyrolysis technique revealed good sensitivity, repeatability and total reversibility towards O_3_ gas of concentrations, 20–1040 ppb [287]. The SEM cross-sectional view and surface morphology of Zn_0.95_Co_0.05_O thin film are shown in figure 10*a*,*b*, respectively. The Co^3+^ improved the gas sensing of ZnO film owing to increase of the interaction sites for target gas. The ozone sensor response of Zn_0.95_Co_0.05_O thin film at 250°C is shown in figure 10*c*. The sensor was found very selective for O_3_ as tested for other gases; NO_2_, NH_3_, and CO as shown in figure 10*d*. The sensing performance was examined for 9 days and observed to be stable. Thus, the Zn_0.95_Co_0.05_O thin film sensor can be used for practical applications as an O_3_ gas sensor.

Excessive O_3_ is a dangerous pollutant emitted from various industrial and household activities. In the past few years, the detection of O_3_ gas has attracted a lot of interest of researchers. The above discussion enables us to conclude that versatile MOSs play a significantly important role in O_3_ detection as well. Again, the metal oxide nanostructures such as core–shell structure, nanospheres and graphene oxide and rGO-based nanocomposites show faster response which is further enhanced by decorating them with noble metals. Thus, in O_3_ detection the MOS materials are also promising candidates for commercial use.

Recently, some another methods are also being developed to detect the O_3_ gas which includes: photoacoustic [293–296], optical O_3_ gas sensors [297–299] O_3_ gas sensors. Keeratirawee *et al.* [293] reported photoacoustic detection of O_3_ gas with a red laser diode using the absorption band. The sensor exhibited about 1.6 ppm LOD for an optical power of the laser diode of about 130 mW. The optical O_3_ sensor based on indium oxide nanoparticles were reported by Wang *et al.* [273]. This sensor showed low LOD of 10 ppb towards O_3_ gas at room temperature. Wu *et al*. [298] prepared optical O_3_ gas sensors by using TiO_2_–WO_3_ composites. The sensor exhibited a good response (23.8) and response/recovery times (155/235 s) towards 2.5 ppm O_3_ at room temperature with LOD of 1000 ppb. This type of sensor has many disadvantages such as limited detection range (sensor dynamic range), cross sensitivity, weather effects (e.g. humidity), cost, light emitting diode (LED) as a light source and sensor robustness [300].

## 4. Conclusion

The climate is changing and affecting the various regions around the world. In future, its negative impacts will most likely be much more severe than before. The huge challenge before us is to first stabilize and then reduce the anthropogenic GHG emissions. To overcome this problem, the very first step in this direction is to measure/detect the GHGs emissions precisely in the Earth's atmosphere and for this purpose, we must have reliable measurement systems for the GHGs. The related data acquisition techniques demand innovative, cost effective, robust and accurate sensors. However, the area of sensors is emerging swiftly of which NMOS-based sensors are paid the utmost attention because of the fulfilment of all these requirements. This review article presents a comprehensive study of the role of NMOS-based gas sensors in the detection of GHGs and their potential to be used in commercial applications. The NMOS-based gas sensors are found to be highly effective in the detection of various GHGs in the Earth's atmosphere. A wide range of morphologies such as nanorods, nanofibres, nanosheets, nanospheres and core–shell structures, etc. are exhibited by nanostructured metal oxides with an advantage of higher surface area and results in increment in adsorption sites available for sensing GHGs. The nanostructured composites with novel materials such as graphene, graphene oxide and rGO make them highly promising candidates for gas sensing applications. Further improvement in their sensing characteristics can be made by doping of noble metals such as Au, Pt and Pd in NMOS. A variety of simple and economical methods are available for the synthesis of NMOS-based nanomaterials which facilitates the tuning of sensing parameters as well as commercial use. Many challenges such as reduction in operating temperature, high selectivity, stability in different measuring environments and longer life time to design suitable NMOS-based GHGs sensors are still open for the enthusiastic researchers in this field.

## Data accessibility

This article has no additional data.
